# Shengxian decoction modulates gut microbiota and microbial metabolism in rats with chronic heart failure

**DOI:** 10.3389/fmicb.2026.1791537

**Published:** 2026-05-20

**Authors:** Hong-jing Li, Fan Gao, Hao-ran Shen, Sheng-yun Gao, Xiao-jing Qin, Ze-qi Yang, Qiu-hong Guo

**Affiliations:** 1 Graduate School of Hebei University of Traditional Chinese Medicine, Hebei, China; 2 The First Affiliated Hospital of Hebei University of Traditional Chinese Medicine, Hebei, China; 3 Hebei University of Traditional Chinese Medicine, Hebei, China

**Keywords:** 16S rRNA gene sequencing, chronic heart failure, fecal metabolomics, gut microbiota, Shengxian decoction

## Abstract

**Background:**

Chronic heart failure (CHF) is a complex syndrome characterized by high morbidity and mortality, imposing a substantial global health burden. Shengxian Decoction (SXT) is a Traditional Chinese Medicine formulation that has demonstrated efficacy in treating CHF. However, its mechanism for modulating the gut microbiota in transverse aortic constriction (TAC)-induced CHF rats remains unclear.

**Methods:**

This study identified components of SXT using liquid chromatography-mass spectrometry (LC–MS). A CHF model was established in rats via TAC surgery. Cardiac function was assessed by echocardiography, and hematological parameters were subsequently analyzed. Myocardial and colonic histopathology were examined by H&E staining, and myocardial fibrosis was evaluated using Masson’s trichrome staining. Intestinal barrier function was assessed using immunohistochemistry (IHC), Western blotting, and RT-qPCR analyses. Fecal 16S rRNA gene sequencing and ultra-performance liquid chromatography-mass spectrometry (UPLC–MS) metabolomics were performed across groups to characterize the gut microbiota and its associated metabolites. Furthermore, MetOrigin and Spearman’s correlation analysis were employed to characterize the associations between gut microbiota and metabolites.

**Results:**

In total, 147 components were identified in SXT. SXT treatment improved cardiac function in CHF rats and reduced serum levels of N-terminal pro-B-type natriuretic peptide (NT-proBNP), lipopolysaccharide (LPS), tumor necrosis factor-α (TNF-α), and interleukin-6 (IL-6). Furthermore, SXT ameliorated pathological alterations in myocardial and colonic tissues and restored the expression of occludin and ZO-1 in colonic tissue. Among them, the high-dose SXT group showed the most prominent therapeutic effect. 16S rRNA sequencing revealed that SXT ameliorated gut microbiota dysbiosis, identifying 17 differentially abundant bacterial genera. Among these, SXT increased the abundance of genera capable of producing short-chain fatty acids. Fecal metabolomic analysis identified 27 differential metabolites. After SXT treatment, levels of glycocholic acid, cholic acid, chenodeoxycholic acid, and prostaglandin A2 were elevated, whereas palmitic acid and metanephrine were reduced. Metabolite tracing analysis indicated that primary bile acid biosynthesis is a key host-microbe co-metabolic pathway. Spearman correlation analysis revealed significant associations between differential gut bacterial genera and differential metabolites.

**Conclusion:**

This study demonstrates the therapeutic efficacy of SXT in alleviating CHF and its potential to regulate microbial–host co-metabolism. These findings provide new insights into the mechanisms underlying the effects of SXT in CHF.

## 1 Introduction

Chronic heart failure (CHF) is the final stage of cardiovascular disease and represents a complex clinical syndrome (Bozkurt et al., 2021; Li et al., 2025a). As one of the diseases with high global morbidity and mortality, CHF currently affects approximately 64 million people worldwide. The prevalence of CHF is projected to increase by approximately 25% by 2030 (Savarese et al., 2022; Tayal et al., 2017). Modern medicine has continuously advanced the treatment of heart failure, including beta-blockers, angiotensin-converting enzyme inhibitors, angiotensin receptor blockers, aldosterone receptor antagonists, and sodium-glucose cotransporter-2 inhibitors. However, the incidence and hospitalization rates of heart failure remain elevated (Heidenreich et al., 2022).

Shengxian decoction (SXT), one of the most well-known formulations in traditional Chinese medicine (TCM), comprises five medicinal materials: Astragali Radix (Huangqi), Bupleuri Radix (Chaihu), Cimicifugae Rhizoma (Shengma), Anemarrhenae Rhizoma (Zhimu), and Platycodonis Radix (Jiegeng). These medicinal materials have demonstrated therapeutic potential in preventing and treating cardiovascular diseases (Huang C. Y. et al., 2021). As the principal herb of SXT, Astragali Radix has been widely reported to exert cardioprotective effects (Li et al., 2022). Astragaloside IV exerts cardioprotective effects in CHF by suppressing inflammatory responses and improving myocardial energy metabolism (Wang et al., 2024). Moreover, various active ingredients in Astragali Radix exert protective effects on the intestine, with underlying mechanisms involving anti-inflammatory activity, immunomodulation, and restoration of intestinal barrier function (Liang H. et al., 2024; Wei et al., 2024). In addition, most of the active ingredients in Astragali Radix have no obvious toxic side effects (Li et al., 2022). A meta-analysis further demonstrated that Astragali Radix injection combined with conventional therapy enhances cardiac function in CHF patients (Han et al., 2024). Studies have shown that timosaponin B-II from Anemarrhenae Rhizoma attenuates LPS-induced cardiomyocyte injury (Xue et al., 2021), while saikosaponins from Bupleuri Radix regulate gut microbiota composition and exhibit myocardial antioxidant activity (Wang et al., 2025; Ding et al., 2019). Ferulic acid and isoferulic acid derived from Cimicifugae Rhizoma protect both the myocardium and microvasculature (Serreli et al., 2021). Metabolomic analysis revealed that Platycodonis Radix in SXT contributes to the synergistic therapeutic effects in CHF rats (Zhang et al., 2014). Collectively, SXT ameliorates CHF through multiple pathways. Researchers propose that early intervention with TCM, together with holistic regulation, may prevent CHF recurrence and reduce adverse effects (Jia et al., 2020).

The gut microbiome is a complex ecosystem of diverse microorganisms present in the human gut that plays a critical role in maintaining internal homeostasis (Ruan et al., 2020). Dysregulation of the abundance and diversity of the gut microbiota can lead to a range of pathological changes, including immune dysfunction, inflammatory responses, and damage to the intestinal barrier (Ruan et al., 2020). These factors can cause or exacerbate other diseases, and the development of heart failure has been demonstrated to be closely associated with gut microbiome dysregulation (Jia et al., 2020). Sun et al. (2022) observed a significant increase in Proteobacteria and a notable decrease in Firmicutes in patients with CHF compared to healthy controls. Pang et al. (2024) investigated the potential causal relationship between the gut microbiota and heart failure using a two-sample Mendelian randomization study. The results identified that seven bacterial taxa in the gut were significantly associated with the risk of heart failure. Furthermore, heart failure progression is also influenced by gut microbial metabolites such as oxidized trimethylamine, bile acids, and short-chain fatty acids (SCFAs) (Jin et al., 2019). Simadibrata et al. (2023) demonstrated that the abundances of *Actinomycetes*, *Enterococci*, and *Streptococcus* were increased, whereas the abundance of *Firmicutes* was decreased in CHF patients. In addition, the abundance of gut microorganisms that produce SCFAs was also reduced. Therefore, the gut microbiota and its metabolites represent promising therapeutic targets for heart failure (Troseid et al., 2020).

Regulating gut microbiota and its metabolites could be a potential target for TCM treatment of cardiovascular disorders (Che et al., 2022; Li et al., 2025b). According to Cao et al. (2024), the use of 20 different kinds of TCMs increased the clinical effectiveness rate of patients with coronary heart disease by 17.78% overall. Subsequent research revealed that these TCMs can regulate the gut microbiota and its metabolites in the treatment of coronary heart disease. However, the mechanism by which SXT acts on gut microbiota and its metabolites in heart failure has not been reported. Therefore, using 16S rRNA gene sequencing and fecal metabolomics, this study explored whether SXT influences CHF progression by modulating gut microbiota composition and its metabolic profile in CHF rats.

## 2 Materials and methods

### 2.1 Kits and materials

N-terminal pro-B-type natriuretic peptide (NT-proBNP) ELISA kit was purchased from Sangon Biotech Co., Ltd. (Shanghai, China). Rat lipopolysaccharide (LPS) ELISA kit was purchased from Jianglai Biotechnology Co., Ltd. (Shanghai, China). The tumor necrosis factor-α (TNF-α) and interleukin-6 (IL-6) ELISA kits were obtained from Wuhan Servicebio Technology Co., Ltd. (Wuhan, China). Trimetazidine hydrochloride tablets (TMZ) were purchased from Servier (Tianjin) Pharmaceutical Co., Ltd. (No. 2019118). Occludin and ZO-1 antibodies were obtained from Affinity Biosciences Co., Ltd. (No. AF5145; No. DF7504), *β*-actin antibody was obtained from Abways Technology Co., Ltd. (No. Ab0035), and horseradish peroxidase (HRP)-conjugated goat anti-rabbit IgG was obtained from Servicebio Technology Co., Ltd. (No. GB23303). Methanol, acetonitrile, ammonia, and isopropanol for UHPLC were purchased from Shanghai Anpu Experimental Technology Co., Ltd. (Shanghai, China). Ammonium acetate and acetic acid for UHPLC were obtained from Shanghai Sigma High Technology Co., Ltd. (Shanghai, China).

### 2.2 Experimental animals

The experimental protocols were approved by the Experimental Animal Care and Use Committee of Hebei University of Traditional Chinese Medicine (Shijiazhuang, China) under Approval Number DWLL202306090. All animal procedures were conducted in strict accordance with internationally recognized guidelines for the Care and Use of Laboratory Animals (NIH Publication No. 85-23, revised 1996) and Ethics Committees in Science: European Perspectives. A total of 36 male Sprague–Dawley (SD) rats, weighing 180–200 g, were obtained from Beijing SiBeiFu Biotechnology Co., Ltd. (Beijing, China) and kept under standard husbandry circumstances (24 ± 4 °C, 12-h light/dark cycle) with free access to food and water for 7 days.

### 2.3 Preparation of SXT

Table 1 summarizes the complete profile of medicinal components in SXT. The accuracy of the plant names was verified using data from http://www.theplantlist.org. The medicinal materials were provided by Anhui Boyao Qiancao National Medicines Co., Ltd. The five medicinal materials were thoroughly mixed and evenly divided into filter bags. The mixture was soaked in 1,000 mL of water for 30 min and then heated to boiling. After the mixture reached boiling, the heat was reduced to maintain a simmering state for 30 min. The mixture was filtered to obtain the primary decoction. 1,000 mL of water was added to the remaining medicinal materials; after boiling, it was simmered for 30 min and filtered to yield the secondary decoction. Both herbal decoctions were mixed and concentrated to obtain extracts with low (0.51 g/mL), medium (1.02 g/mL), and high (2.04 g/mL) concentrations. The concentrated product was aliquoted into sterile containers and stored at 4 °C until use. The dosages of SXT (5.1 g/kg, 10.2 g/kg, 20.4 g/kg) were approximately one, two, and four times the equivalent dose of SXT used clinically in adults, and were converted by normalization of body surface area. The specific doses were 5.1 g, 10.2 g, and 20.4 g of SXT crude herbs per kilogram of rat body weight, respectively. In previous studies, the components of SXT were analyzed by high-performance liquid chromatography (HPLC), which confirmed the stability and reliability of the prepared TCM solution (Yang et al., 2023).

**Table 1:** Chinese medicinal herbs contained in Shengxian decoction and supplier information.

Chinese naming of different ingredients (with their corresponding Latin names)	Full botanical plant names	Amount (g)	Manufacturer source	Batch number
Hang Qi (*Astragali Radix*)	*Astragalus membranaceus* (Fisch.) Bunge.var. *mongholicus* (Bunge) P.K.Hsiao	22.4	Anhui Boyao Qiancao National Medicines Co., Ltd.	2,202,076
Sheng Ma (*Cimicifugae Rhizoma*)	*Cimicifuga heracleifolia* Komar.	3.7	Anhui Boyao Qiancao National Medicines Co., Ltd.	2,202,023
Chai Hu (*Bupleuri Radix*)	*Bupleurum chinense* DC.	5.6	Anhui Boyao Qiancao National Medicines Co., Ltd.	2,202,079
Zhi Mu (*Anemarrhenae Rhizoma*)	*Anemarrhena asphodeloides* Bunge.	11.2	Anhui Boyao Qiancao National Medicines Co., Ltd.	2,202,003
Jie Geng (*Platycodonis Radix*)	*Platycodon grandiflorum* (Jacq.) A.DC.	5.6	Anhui Boyao Qiancao National Medicines Co., Ltd.	2,202,059

### 2.4 LC–MS analysis of SXT

After SXT was prepared as described above, it was concentrated to 50 mL. A 100 μL aliquot of the solution was transferred into a 1.5 mL centrifuge tube, mixed with 300 μL of methanol by vortexing, and subjected to ultrasonic extraction for 1 h. Subsequently, the mixture was centrifuged at 12,000 rpm and 4 °C for 10 min. Finally, 100 μL of the supernatant was transferred into an injection vial for LC–MS analysis. The detailed procedure is provided in the Supplementary material.

### 2.5 Animal grouping and treatment

After 7 days of acclimatization, SD rats were randomly divided into the sham group (*n* = 6) and the model group (*n* = 30). Transverse aortic constriction (TAC) in the model group was performed to establish the CHF model. Specific procedures were carried out as described in the previous study (Yang et al., 2023). The rats were anesthetized with intraperitoneal injection of 3% sodium pentobarbital at a dose of 1 mL/kg, followed by fixation in a supine position on a surgical platform. After hair removal and disinfection of the surgical site, an incision was made along the superior margin of the second rib. The thoracic cavity was opened by blunt dissection of the muscle layers. The thymus and surrounding tissues were carefully separated to expose the aortic arch. A 27-gauge blunt needle (diameter 0.4 mm) was placed parallel to the aortic arch between the brachiocephalic trunk and the left common carotid artery. A 5–0 surgical suture was passed under the aortic arch to ligate both the aorta and the needle. After secure ligation, the needle was removed. The thoracic cavity was closed, and the muscle and skin layers were sutured. Postoperatively, the wound was promptly disinfected, with body temperature maintained and vital signs monitored. The sham group underwent the same procedure without aortic ligation. Stringent measures were implemented throughout the experimental procedures to minimize animal distress. The animals were given unlimited access to food and water for 4 weeks before beginning treatment. Echocardiographic examination performed 4 weeks after surgery showed that the left ventricular ejection fraction (LVEF) in the model group was reduced by more than 25% compared to the sham group. This finding was consistent with previous results (Chen et al., 2024), confirming the successful establishment of the model. Finally, the model group rats were randomly assigned to five groups (*n* = 6), as follows: the model group, the TMZ group (6.3 mg/kg/day), the SXT-L group (5.1 g/kg/day, 1 mL/100 g with 0.51 g/mL SXT), the SXT-M group (10.2 g/kg/day, 1 mL/100 g with 1.02 g/mL SXT), and the SXT-H group (20.4 g/kg/day, 1 mL/100 g with 2.04 g/mL SXT). The sham and model groups were administered 0.9% normal saline by gavage. The other four groups received the corresponding interventions (SXT at different doses or TMZ) by gavage. All treatments were administered by gavage for 8 weeks.

### 2.6 Echocardiography

Before sample collection, the rats were anesthetized with 5% isoflurane in oxygen, and their chest hair was removed with a depilator. Echocardiography was performed using an MS-250 probe (Vevo 2100, Visual Sonics Inc., Toronto, Canada). The operating table was adjusted along the X- and Y-axes to obtain a parasternal long-axis B-mode view. After clear visualization of the left ventricular and aortic structures, the probe and operating table were fixed in position, and the probe angle was further adjusted to acquire the parasternal long-axis M-mode images. LVEF and left ventricular fractional shortening (LVFS) were measured using the long-axis measurement method (PLAX). For each rat, three cardiac cycles were selected for analysis, and the mean value was calculated. All measurements were performed by examiners blinded to the group assignments.

### 2.7 Sample collection

After 8 weeks of treatment, all rats were anesthetized with 3% pentobarbital sodium. The rats were placed on a sterile operating table. Blood from the femoral artery was collected and allowed to stand at room temperature for 4 h, followed by centrifugation at 3,000 rpm and 4 °C for 15 min. After the serum was collected, it was stored at −80 °C for subsequent analysis. After dissection, the colon and heart tissues were collected and fixed in 4% paraformaldehyde, while fresh fecal samples were placed in cryotubes and stored at −80 °C for subsequent analysis.

### 2.8 Determination of serum indices

According to the manufacturer’s instructions, serum levels of NT-proBNP, LPS, TNF-α, and IL-6 were determined using the corresponding assay kits to evaluate the severity of heart failure and systemic inflammatory status.

### 2.9 Histopathological analysis

Heart and colon tissues were fixed in 4% paraformaldehyde, followed by dehydration, clearing, and paraffin embedding. The paraffin-embedded blocks were sectioned using a microtome, and the sections were mounted onto glass slides and flattened. After deparaffinization, the sections were stained with hematoxylin and eosin (H&E) and examined under a Leica DM5000B microscope. Masson’s trichrome staining was performed to assess myocardial fibrosis. Under the microscope, collagen fibers appeared blue, whereas normal myocardium appeared red. The fibrotic area was quantified using ImageJ software.

### 2.10 Immunohistochemical (IHC) staining

The expression of occludin and ZO-1 proteins in colonic tissues was evaluated by IHC. After deparaffinization and dehydration through a graded ethanol series, sections were incubated with 3% hydrogen peroxide to inactivate endogenous enzymes. Antigen retrieval was performed with a high-pH antigen retrieval solution, followed by blocking with 5% BSA. Sections were incubated overnight at 4 °C with the corresponding primary antibodies. After washing, sections were incubated with secondary antibodies for 30 min at room temperature. Following Diaminobenzidine (DAB) staining, nuclei were counterstained with hematoxylin. Finally, the sections were mounted with neutral balsam and imaged under a light microscope. The acquired images were analyzed with ImageJ software to quantify the positive staining intensity of occludin and ZO-1.

### 2.11 16S rRNA gene sequencing analysis

#### 2.11.1 DNA extraction and PCR amplification

16S rRNA gene sequencing was employed to characterize the gut microbiota composition in fecal samples collected from rats in each group. The OMEGA Soil DNA Kit (M5635-02, Omega Bio-Tek, Norcross, GA, USA) was used to extract total genomic DNA. The quality and quantity of extracted DNA were assessed using 0.8% agarose gel electrophoresis and a NanoDrop NC200 spectrophotometer (Thermo Fisher Scientific, Waltham, MA, USA). Polymerase chain reaction (PCR) was performed to amplify the V3-V4 region of the bacterial 16S rRNA gene using the primers 338F (5′-barcode + ACTCCTACGGGAGGCAGCA-3′) and 806R (5′-GGACTACHVGGGTWTCTAAT-3′). To distinguish samples, a unique 7-bp barcode was incorporated into the primers. The amplification mixtures included 5 μL of 5 × reaction buffer, 5 μL of 5 × High-Fidelity GC buffer, 0.25 μL of High-Fidelity DNA Polymerase, 2 μL of 2.5 mM dNTPs, 1 μL of each forward and reverse primer at 10 μM, 2 μL of DNA template, and 8.75 μL of ddH_2_O. The PCR amplification protocol consisted of an initial denaturation at 98 °C for 3 min, followed by 26 cycles of denaturation at 98 °C for 30 s, annealing at 52 °C for 30 s, and extension at 72 °C for 45 s, with a final extension at 72 °C for 5 min. After amplification, the reaction mixtures were held at 12 °C, and the PCR products were purified using Vazyme VAHTS™ DNA Clean Beads.

#### 2.11.2 DNA library construction and sequencing

The library was constructed using the Illumina TruSeq Nano DNA LT Library Prep Kit. Library quality assessment and quantification were performed using the Agilent Bioanalyzer instrument with Agilent High Sensitivity DNA Kit and Promega QuantiFluor fluorometer with Quant-iT PicoGreen dsDNA Assay Kit. Finally, 2 × 250 bp paired-end sequencing was performed on the Illumina NovaSeq platform using the NovaSeq 6000 SP Reagent Kit (500 cycles) at Shanghai Personal Biotechnology Co., Ltd.

#### 2.11.3 Processing of sequencing data

The raw sequence data were demultiplexed using the demux plugin, followed by primer cutting with the cutadapt plugin. Next, quality filtering, denoising, and chimera removal were performed using the DADA2 plugin. The denoised sequences were used to generate amplicon sequence variants (ASVs) and a feature table. To mitigate the effect of sequencing depth on *α*- and *β*-diversity analyses, the sequence counts of each sample were rarefied to a depth of 16,869. Bacterial taxonomic annotation was assigned by comparing ASVs against the Greengenes2 (2022.10) database using the classify-sklearn naïve Bayes classifier in the QIIME2 feature classifier plugin (Bokulich et al., 2018; Kõljalg et al., 2013).

#### 2.11.4 Bioinformatics analyses

Data analysis was performed using QIIME2 and R (v3.6.0). *α*-diversity indices, including the Chao1 and Shannon indices, were compared among groups via Kruskal–Wallis test. Results were visualized using the R package “ggplot2.” *β*-diversity was calculated based on the Bray–Curtis distance matrix and visualized through principal coordinate analysis (PCoA) and non-metric multidimensional scaling (NMDS) (Ramette, 2007). Venn diagrams were generated using the R package “VennDiagram” to illustrate shared and unique ASVs among different sample groups (Zaura et al., 2009). The “qiime taxa barplot” command was used to visualize the taxonomic composition across different taxonomic levels in each sample. Linear discriminant analysis of effect size (LEfSe) was applied with default parameters to identify microbial taxa exhibiting significant differences in abundance among groups (LDA > 2 and *p* < 0.05) (Segata et al., 2011). Functional prediction of the microbial community was performed using the Phylogenetic Investigation of Communities by Reconstruction of Unobserved States (PICRUSt2) algorithm, with prediction results annotated using the MetaCyc^1^ and KEGG^2^ databases (Douglas et al., 2020).

### 2.12 Metabolomics analysis

#### 2.12.1 Sample preparation

First, 25 mg of fecal sample was combined with microspheres and 500 μL of extract solvent (MeOH: ACN: H_2_O, 2:2:1 (v/v)), and then vortexed for 30 s. The mixed sample was homogenized at 35 Hz for 4 min, and then transferred to an ice water bath for sonication (5 min, repeated 3 times). The samples were placed at −40 °C for 1 h and then centrifuged at 12,000 rpm and 4 °C for 15 min. The supernatant was transferred to the injection vial for online detection. Equal aliquots of the supernatant from each sample were pooled to generate quality control (QC) samples.

#### 2.12.2 UPLC–MS/MS detection

UPLC–MS/MS analyses were performed using a UHPLC system (Vanquish, Thermo Fisher Scientific) with a Waters ACQUITY UPLC BEH Amide column (2.1 mm × 50 mm, 1.7 μm) coupled to an Orbitrap Exploris 120 mass spectrometer (Orbitrap MS, Thermo) (Wang et al., 2016). The mobile phase consisted of 25 mmol/L ammonium acetate and 25 mmol/L ammonium hydroxide in water (pH = 9.75) (phase A) and acetonitrile (phase B). The injector temperature was 4 °C, and the injection volume was 2 μL. The Orbitrap Exploris 120 mass spectrometer could perform primary and secondary mass spectrometry data acquisition controlled by Xcalibur software. The parameters were set as follows: sheath gas flow rate, 50 Arb; aux gas flow rate, 15 Arb; capillary temperature, 320 °C; full MS resolution, 60,000; MS/MS resolution, 15,000; collision energy, NCE 20/30/40; and spray voltage, 3.8 kV in positive mode and −3.4 kV in negative mode. In this study, 8,669 and 6,195 chromatographic peaks were detected in the positive and negative ion modes, respectively, from which 1,336 and 900 metabolites were identified.

#### 2.12.3 Data analysis

The raw data were converted to mzXML format using ProteoWizard. XCMS was then used for peak alignment, retention time correction, and peak area extraction. The data were subsequently filtered and searched against both a locally constructed database and public databases (Luo et al., 2017; Gu et al., 2018). Structural identification of metabolites in biological samples was achieved by matching their retention times, molecular masses (mass error < 10 ppm), MS/MS spectra, and collision energies to entries in reference databases. All identifications were rigorously verified and confirmed through a secondary manual review. Multivariate statistical analysis was performed using the R package “ropls,” including principal component analysis (PCA) and orthogonal partial least squares-discriminant analysis (OPLS-DA). Subsequently, the OPLS-DA model was evaluated for its robustness and reliability through 200 permutation tests. Furthermore, variable importance in projection (VIP) values were calculated using OPLS-DA, and differential metabolites between groups were identified based on VIP > 1 and *p* < 0.05 (Student’s *t*-test). Pathway enrichment analysis of the differential metabolites was performed using the MetaboAnalyst 4.0 platform. Traceability analysis of differential metabolites was carried out using the MetOrigin platform. Functional analysis, source analysis, and Sankey diagram construction were conducted using the basic analysis mode on the official website (Li et al., 2024).

### 2.13 Correlation profiling between metabolites and gut microbiota

Pearson correlation analysis was performed to assess the relationships among the differential metabolites identified in fecal metabolomics, the differential bacterial genera obtained by 16S rRNA gene sequencing, and clinical indicators. The screening criteria were set as a correlation coefficient |*r*| > 0.6 and *p* < 0.05. The correlations between metabolites and clinical indicators, bacterial genera and clinical indicators, and metabolites and bacterial genera were visualized as heatmaps using the ggplot2 package in R (*p < 0.05, **p < 0.01). Red indicates positive correlations, while blue indicates negative correlations.

### 2.14 Real-time quantitative polymerase chain reaction (RT-qPCR) analysis

Total RNA was extracted from rat colonic tissue using an ultrapure RNA extraction kit. RNA purity and concentration were measured, followed by reverse transcription for cDNA synthesis. The PCR reaction was performed in a 20 μL volume under the following conditions: initial denaturation at 95 °C for 30 s, followed by 40 cycles of denaturation at 95 °C for 15 s, annealing at 60 °C for 30 s, and extension at 60 °C for 30 s. Relative expression levels of the target genes were calculated using the 2^−ΔΔCt^ method, with *β*-actin as the internal control. The primer sequences are listed in Table 2.

**Table 2:** Primer sequences for real-time quantitative polymerase chain reaction.

Target gene		Primer sequence (5′ → 3′)
Occludin	Forward	CGGCAAAGTGAATGGCAAGA
	Reverse	TCATCCACGGACAAGGTCAGAG
ZO-1	Forward	CGGGCTACCTTATTGAATGTCC
	Reverse	GAGCGAACTGAATGGTCTGATG
*β*-actin	Forward	TGCTATGTTGCCCTAGACTTCG
	Reverse	GTTGGCATAGAGGTCTTTACGG

### 2.15 Western blot analysis

Total protein was extracted from colonic tissue using RIPA lysis buffer. The tissue was homogenized, and the samples were incubated on ice for 30 min, followed by centrifugation at 12,000 rpm for 10 min at 4 °C. The supernatant was collected, and protein concentration was determined using a BCA assay. Equal amounts of protein from each group were separated by sodium dodecyl sulfate–polyacrylamide gel electrophoresis (SDS–PAGE). Proteins were transferred onto PVDF membranes. The membranes were blocked with 5% milk solution for 2 h and then incubated overnight at 4 °C with occludin (1:2,000) and ZO-1 (1:1,000) antibodies. After washing, membranes were incubated with appropriate secondary antibodies at 37 °C for 1 h. Finally, protein band intensities were analyzed and quantified using ImageJ software.

### 2.16 Statistical analysis

Statistical analyses were performed using SPSS 26.0 software. Data are presented as mean ± standard deviation (SD). One-way analysis of variance (ANOVA) was used to assess differences among groups, with *post-hoc* pairwise comparisons conducted using the LSD test. For data exhibiting non-normal distribution or heteroscedasticity, the Kruskal–Wallis test with Dunn’s *post-hoc* test was used for nonparametric analysis. Pearson correlation analysis was performed using R software. Graphs were generated using GraphPad Prism version 8.4. In all statistical analyses, *p* < 0.05 was considered statistically significant, while *p* < 0.01 was regarded as highly statistically significant.

## 3 Results

### 3.1 Pharmacodynamic evaluation

#### 3.1.1 SXT improves cardiac function in rats with TAC-induced CHF

In this study, the chemical components of SXT were analyzed by LC-MS. Total ion chromatograms in both positive and negative ion modes revealed that the chemical components of SXT were complex and diverse (Supplementary Figure S1). By comparing m/z values, retention times, and MS/MS fragmentation patterns with reference literature and databases, 147 chemical components were preliminarily identified. Representative components are listed in Supplementary Table S2. The results indicated that the identified chemical components were highly consistent with the botanical origins of the medicinal materials in SXT, primarily including flavonoids, phenolic acids, and other characteristic components. The identification of these chemical components provided a material basis for subsequent mechanistic investigations of SXT.

**Figure 1:**
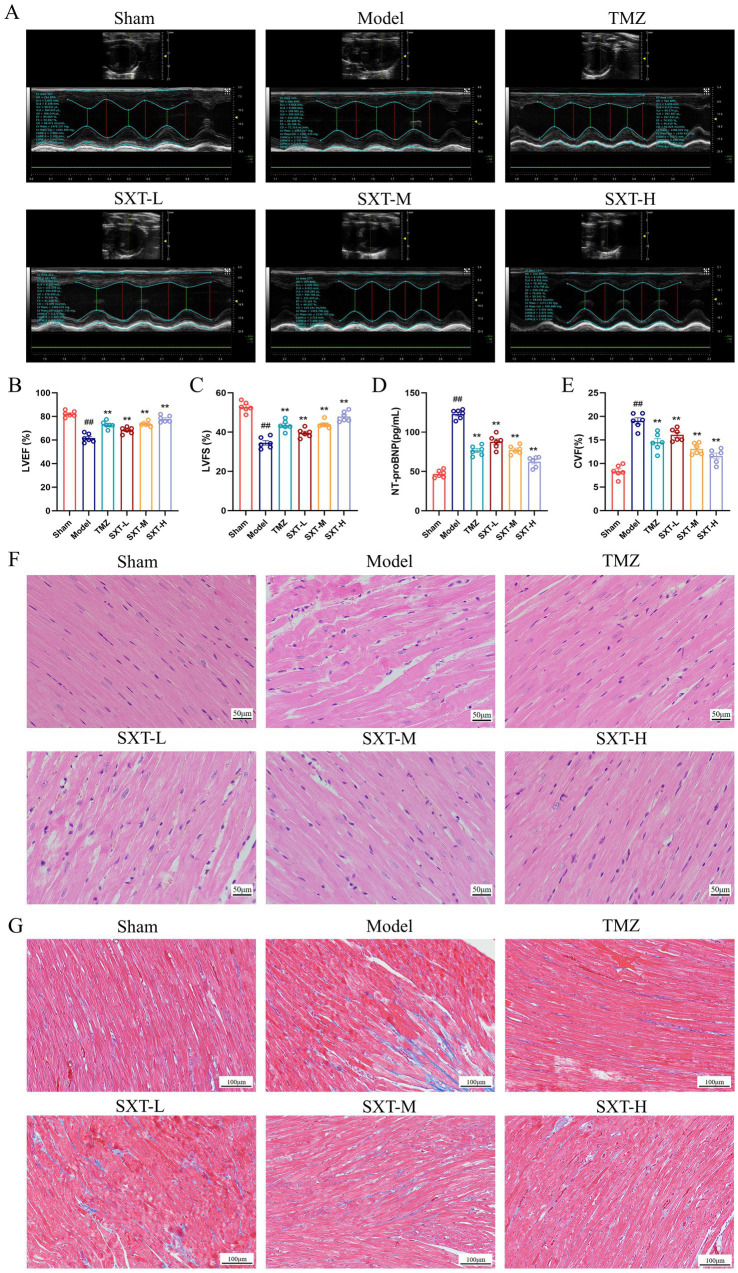
**(A)** Representative echocardiographic images of the six groups. Cardiac function data on LVEF **(B)** and LVFS **(C)**. **(D)** Serum NT-proBNP levels measured by ELISA. **(E)** Collagen volume fraction. **(F)** Representative images of myocardial tissue stained with H&E (scale bar = 50 μm). **(G)** Representative images of myocardial tissue stained with Masson (scale bar = 100 μm). ^##^*p* < 0.01 vs. the sham group; ^**^p < 0.01 vs. the model group.

Representative echocardiographic images are shown in Figure 1A. Compared with the sham group, LVEF and LVFS were reduced in the model group (*p* < 0.01), indicating impaired cardiac function in CHF rats. After SXT or TMZ treatment, LVEF and LVFS were increased compared with the model group (*p* < 0.01). These findings suggest that cardiac function was improved after treatment. Notably, the SXT-H or TMZ groups showed significantly increased LVEF levels (Figures 1B,C). Compared with the sham group, serum NT-proBNP levels were increased in the model group (*p* < 0.01), indicating impaired cardiac function. After treatment with SXT or TMZ, serum NT-proBNP levels were reduced (*p* < 0.01). However, the SXT-H group had notably decreased NT-proBNP levels (Figure 1D). H&E staining showed that myocardial fibers in the sham group were well organized with clearly visible nuclei. In contrast, the model group exhibited disrupted and disorganized myocardial fibers with loss of nuclear integrity. Treatment with SXT or TMZ restored myocardial architecture and reduced necrosis (Figure 1F). Masson’s trichrome staining revealed that, compared with the sham group, collagen deposition was significantly increased in the model group (*p* < 0.01). However, treatment with SXT or TMZ significantly reduced the extent of myocardial fibrosis (*p* < 0.01) (Figures 1E,G).

Collectively, the model group exhibited significant cardiac dysfunction, elevated NT-proBNP levels, and myocardial injury, consistent with previous reports (Yang et al., 2023; Chen et al., 2024), confirming the successful establishment of the TAC-induced model. These pathological changes were alleviated to varying degrees following treatment with SXT or TMZ.

#### 3.1.2 SXT alleviates gut dysfunction in rats with TAC-induced CHF

**Figure 2:**
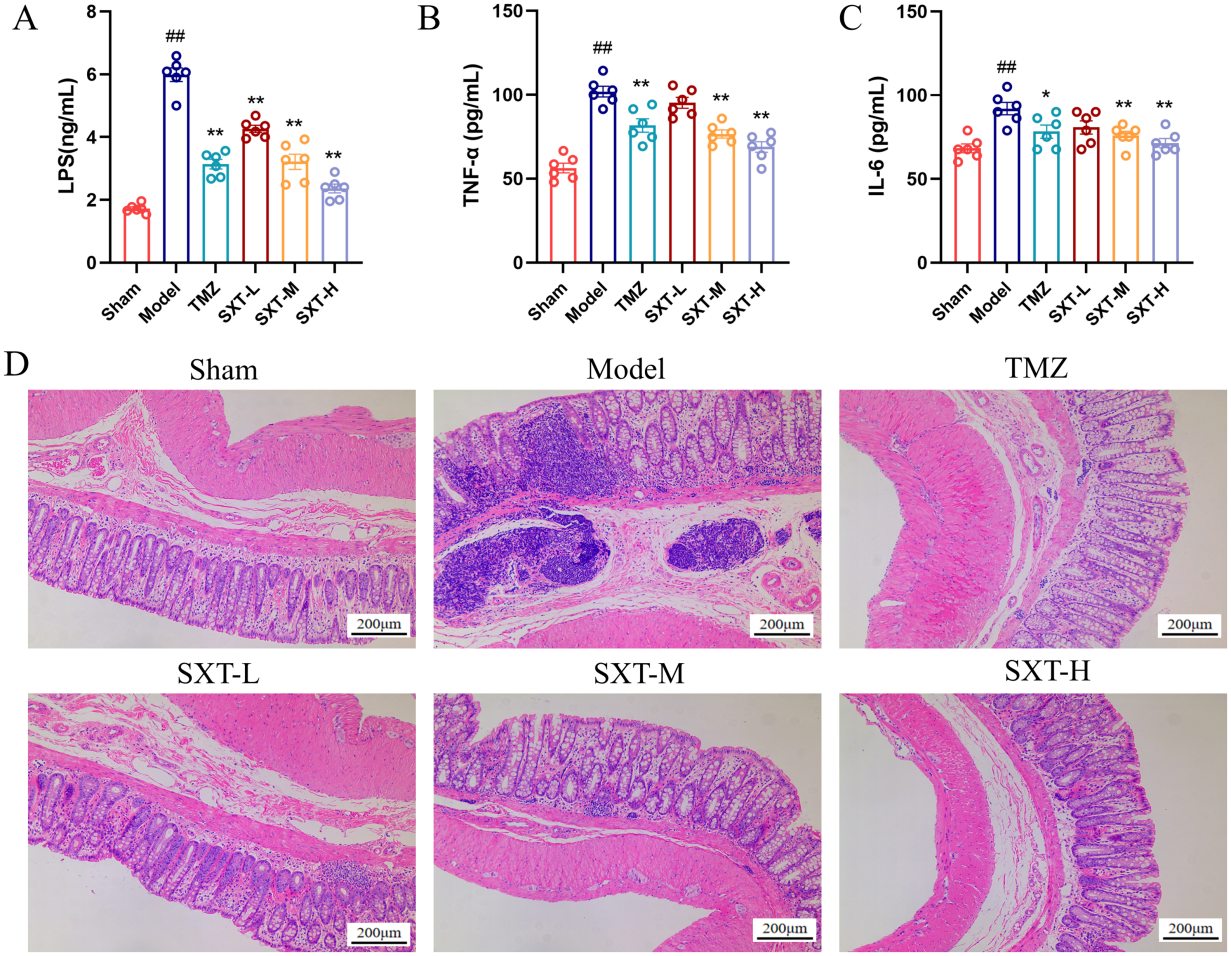
Serum levels of LPS **(A)**, TNF-α **(B)**, and IL-6 **(C)** measured by ELISA. **(D)** Representative images of colonic tissue stained with H&E (scale bar = 200 μm). Notes: ^##^*P* < 0.01 vs. the sham group; *P < 0.05, **P < 0.01 vs. the model group.

Serum levels of LPS, TNF-α, and IL-6 were measured in each group. Compared with the sham group, serum levels of LPS, TNF-α, and IL-6 were significantly elevated (*p* < 0.01) in the model group, indicating impaired intestinal barrier function and accompanied by systemic inflammatory responses. After treatment with SXT or TMZ, these indicator levels were significantly reduced (*p* < 0.01), with the most pronounced effect observed in the SXT-H group (Figures 2A–C). H&E staining of colonic tissue revealed intact intestinal architecture with regularly arranged villi in the sham group. However, the model group exhibited disrupted intestinal architecture with inflammatory cell infiltration in the mucosa and submucosa. SXT treatment restored intestinal structure and reduced inflammatory cell infiltration. The SXT-H treatment restored gut function better than the SXT-L and SXT-M treatments. The intestinal structure in the TMZ group did not fully recover, and inflammatory cells were still seen in the mucosa and submucosa (Figure 2D). These findings suggest that SXT ameliorates intestinal injury and attenuates systemic inflammation in CHF rats.

**Figure 3:**
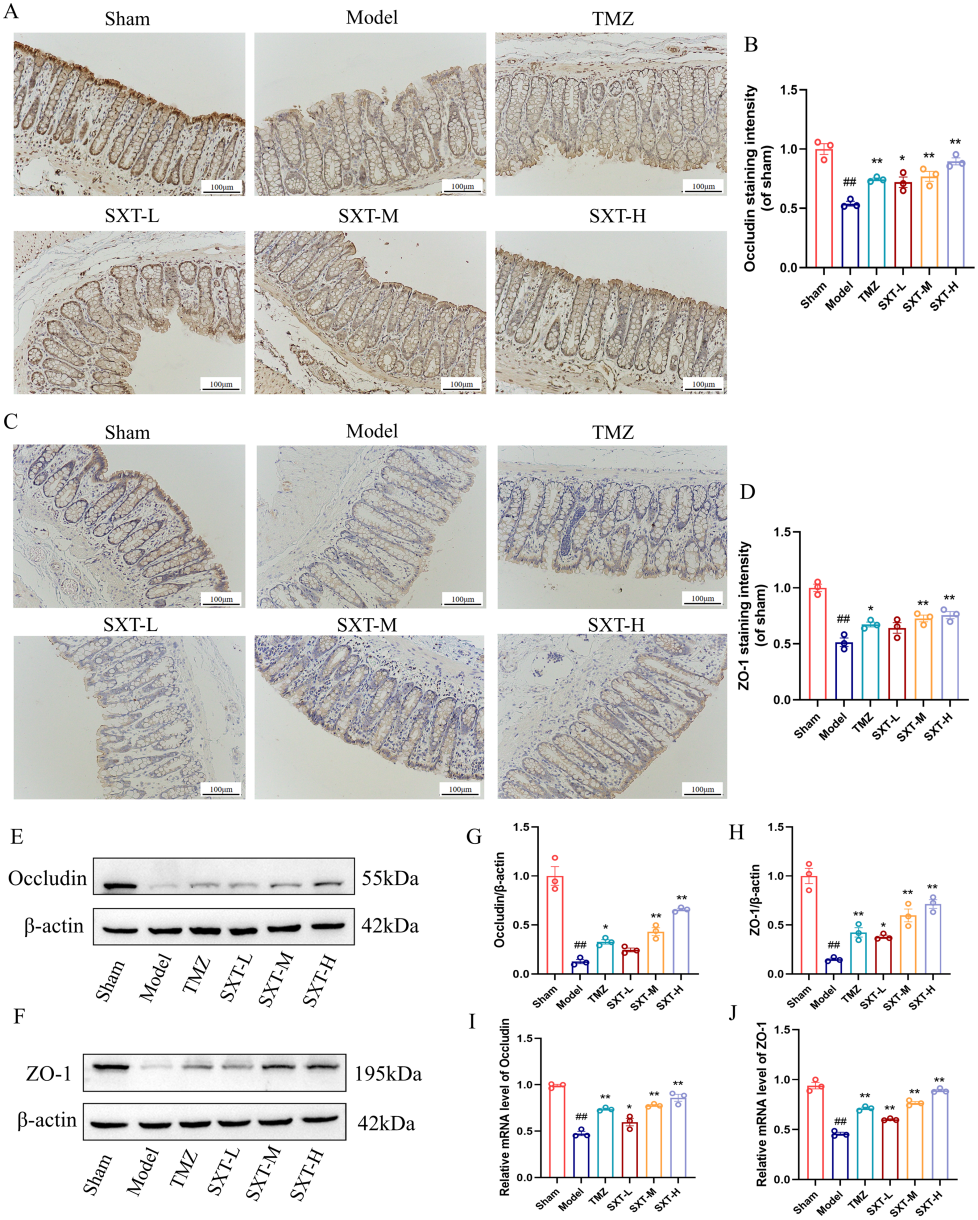
**(A,C)** IHC staining for occludin and ZO-1 (scale bar = 100 μm). **(B,D)** Positive staining intensity of occludin and ZO-1. **(E,F)** Protein expression levels of occludin and ZO-1. **(G,H)** Quantitative analysis of occludin and ZO-1 expression. **(I,J)** Relative mRNA expression levels of occludin and ZO-1. ^##^*p* < 0.01 vs. the sham group; ^*^p < 0.05, ^**^p < 0.01 vs. the model group.

The tight junction proteins occludin and ZO-1 are critical indicators of intestinal barrier integrity. IHC analysis revealed that, compared with the sham group, occludin and ZO-1 expression was significantly reduced in the colon of model rats, indicating intestinal barrier disruption. Treatment with SXT or TMZ significantly increased the expression of both proteins (Figures 3A–D). The expression levels of occludin and ZO-1 in rat colonic tissues were further examined by Western blot and RT-qPCR. Compared with the sham group, the protein and mRNA levels of occludin and ZO-1 were significantly decreased in the model group. In contrast, SXT or TMZ treatment significantly increased the protein and mRNA levels of occludin and ZO-1. These findings were consistent with the IHC results, further confirming that SXT can improve intestinal barrier function (Figures 3E–J).

These findings suggest that SXT protects both cardiac and gut function in a potentially dose-dependent manner. However, our previous study found that the liver function of the SXT-H group was normal, indicating that this dose is safe and reliable (Yang et al., 2023). Consequently, 20.4 g/kg was selected for subsequent analyses, including 16S rRNA sequencing and fecal metabolomics.

### 3.2 16S rRNA sequencing results

#### 3.2.1 Preprocessing and quality control of sequencing data

**Figure 4:**
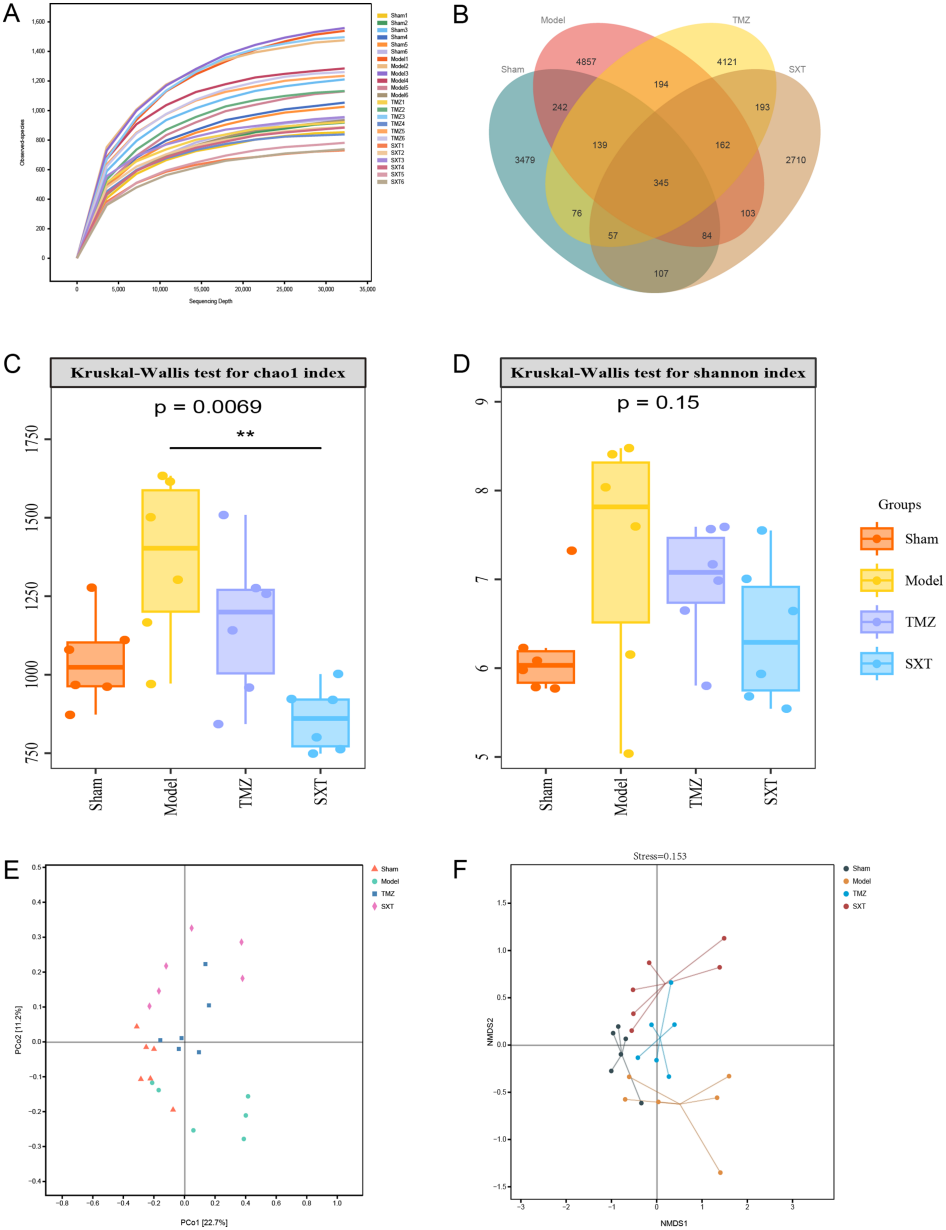
**(A)** Rarefaction curve of the four groups. **(B)** Venn diagram of the four groups. **(C)** Chao1 index of the four groups. **(D)** Shannon index of the four groups. **(E)** PCoA plot of the four groups. **(F)** NMDS plot of the four groups. The Chao1 and Shannon indices were analyzed using the Kruskal–Wallis test for multiple-group comparisons, followed by Dunn’s *post-hoc* test for pairwise comparisons (*n* = 6). ^##^*p* < 0.01 vs. the Sham group; ^**^p < 0.01 vs. the model group.

The V3-V4 region of the 16S rRNA gene was sequenced in four groups of rats. A total of 2,139,790 high-quality sequences were obtained from 24 samples, with an average of 89,158 ± 8,809 tags per sample, representing 17,216 ASVs. The rarefaction curve approached a plateau and extended toward the right end of the x-axis with increasing sequencing depth, indicating that the sequencing data were sufficient and suitable for subsequent analysis (Figure 4A).

#### 3.2.2 Effect of SXT on gut microbiota diversity in CHF rats

The Venn diagrams were used to identify shared and unique species across groups. The shared species are present in most samples, and the unique species may be the species that cause differences between groups. A total of 4,529, 6,126, 3,761, and 5,287 ASVs were identified in the sham, model, SXT, and TMZ groups, respectively. The number of ASVs was increased in TAC-induced CHF rats compared with the sham group, whereas SXT or TMZ treatment reduced the ASV number, bringing it closer to the level observed in the sham group. Among these, 345 ASVs were shared across all groups, representing the core microbiota. In addition, 3,479, 4,857, 2,710, and 4,121 ASVs were unique to the sham, model, SXT, and TMZ groups, respectively (Figure 4B). The number of unique ASVs followed the order: model > TMZ > sham > SXT, indicating that microbial communities in the SXT and TMZ groups partially recovered after treatment. *α*-diversity indices are commonly used to assess microbial diversity and richness. The Chao1 and Shannon indices were calculated to estimate species richness and diversity.

Compared with the sham group, the model group showed increased Chao1 and Shannon indices, but the differences were not statistically significant. After SXT treatment, the Chao1 index was significantly reduced compared with the model group (*p* < 0.01), whereas the Shannon index showed no significant change. In the TMZ group, neither index differed significantly from the model group (Figures 4C,D). Collectively, SXT treatment could reverse the increase in species richness but had little effect on species diversity. In addition, the effect of TMZ was less pronounced than that of SXT.

#### 3.2.3 Effect of SXT on gut microbiota structure in CHF rats

*β*-diversity reflects differences in species composition across samples or environmental gradients. To assess intergroup community differences, principal coordinate analysis (PCoA) based on Bray–Curtis distances, a classic unconstrained ordination method, was performed. This method projects the sample distance matrix into a low-dimensional space to preserve the original distance relationships between samples to the greatest extent. The PCoA results showed that the contributions of principal components PC1 and PC2 reached 22.7 and 11.2%, respectively. Samples from the sham and model groups did not overlap, with most sham samples positioned on the left of the PC1 axis. Furthermore, the distribution of model group samples was distinct from those of the SXT and TMZ groups. After SXT or TMZ treatment, the gut microbial structure was similar to that of the sham group (Figure 4E). Non-metric multidimensional scaling (NMDS) was also applied to assess *β*-diversity. NMDS reduces the dimensionality of the distance matrix to simplify data and reveal sample distribution patterns at specific distance scales. The NMDS results revealed that the samples in the sham group were relatively concentrated, mostly located on the left side of the NMDS1 axis, whereas the model group exhibited a scattered distribution and was distinctly separated from the sham group. Along the NMDS2 axis, group positions from top to bottom were SXT, TMZ, sham, and model, indicating lower similarity of the treatment groups to the model group (Figure 4F). These results indicate that SXT and TMZ may promote the restoration of gut microbial structure in CHF rats.

#### 3.2.4 Analysis of horizontal community across groups

**Figure 5:**
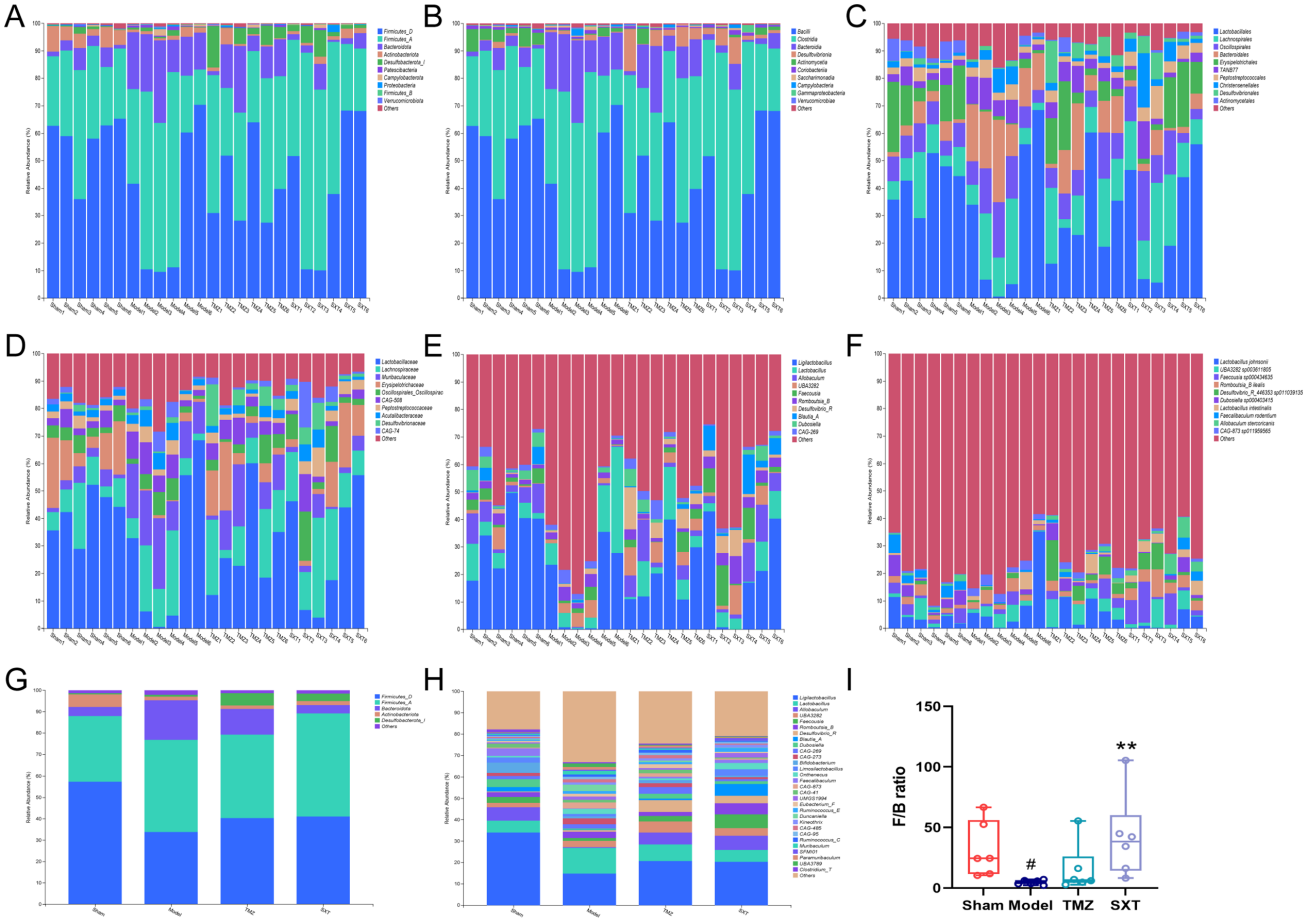
Relative abundance of the top 10 taxa at different levels in four groups. **(A–F)** Relative abundances at the phylum, class, order, family, genus, and species levels. **(G,H)** Microbial community composition at the phylum and genus levels. **(I)** *Firmicutes* / *Bacteroidota* (F/B) ratio across groups. ^#^*p* < 0.05 vs. the sham group; ^**^p < 0.01 vs. the model group.

To study the taxa composition of the fecal microbiota, the relative abundance and proportion of each taxa were displayed using percentage stacked histograms. The top 10 taxa with the highest relative abundance across the four groups were selected for analysis based on the results of the taxa annotation. At the phylum level, the top 10 phyla in abundance were *Firmicutes_D, Firmicutes_A, Bacteroidota, Actinobacteriota, Desulfobacterota_I, Patescibacteria, Campylobacterota, Proteobacteria, Firmicutes_B,* and *Verrucomicrobiota* (Figure 5A). Figures 5B–F display the top 10 taxa for the other levels. At the phylum level, *Firmicutes_D*, *Firmicutes_A*, *Bacteroidota*, *Actinobacteriota*, and *Desulfobacterota_I* were the most abundant across all groups. Relative abundances of *Firmicutes_D* followed the order: sham group > SXT group > TMZ group > model group, accounting for 57.30, 41.08, 40.38, and 33.93%, respectively. Relative abundances of *Firmicutes_A* were ordered: SXT group > model group > TMZ group > sham group, with proportions of 48.25, 43.02, 38.98, and 30.68%, respectively. *Bacteroidota* relative abundances followed the order: model group > TMZ group > sham group > SXT group, representing 18.46, 11.99, 4.26, and 3.77%, respectively. The relative abundances of *Actinobacteriota* followed the sequence sham group > SXT group > model group > TMZ group, accounting for 5.88, 1.85, 1.64, and 1.60%, respectively. *Desulfobacterota_I* relative abundances were ordered: TMZ group > SXT group > model group > sham group, with proportions of 5.77, 3.62, 0.85, and 0.64%, respectively (Figure 5G, Supplementary Table S1). *Bacteroidota* and *Firmicutes* were the most prevalent in all samples. In the model group, *Firmicutes* relative abundance was significantly reduced, *Bacteroidota* relative abundance was significantly increased, and the Firmicutes/Bacteroidota (F/B) ratio decreased (*p* < 0.05). Notably, SXT treatment increased the F/B ratio (*p* < 0.01) (Figure 5I).

**Figure 6:**
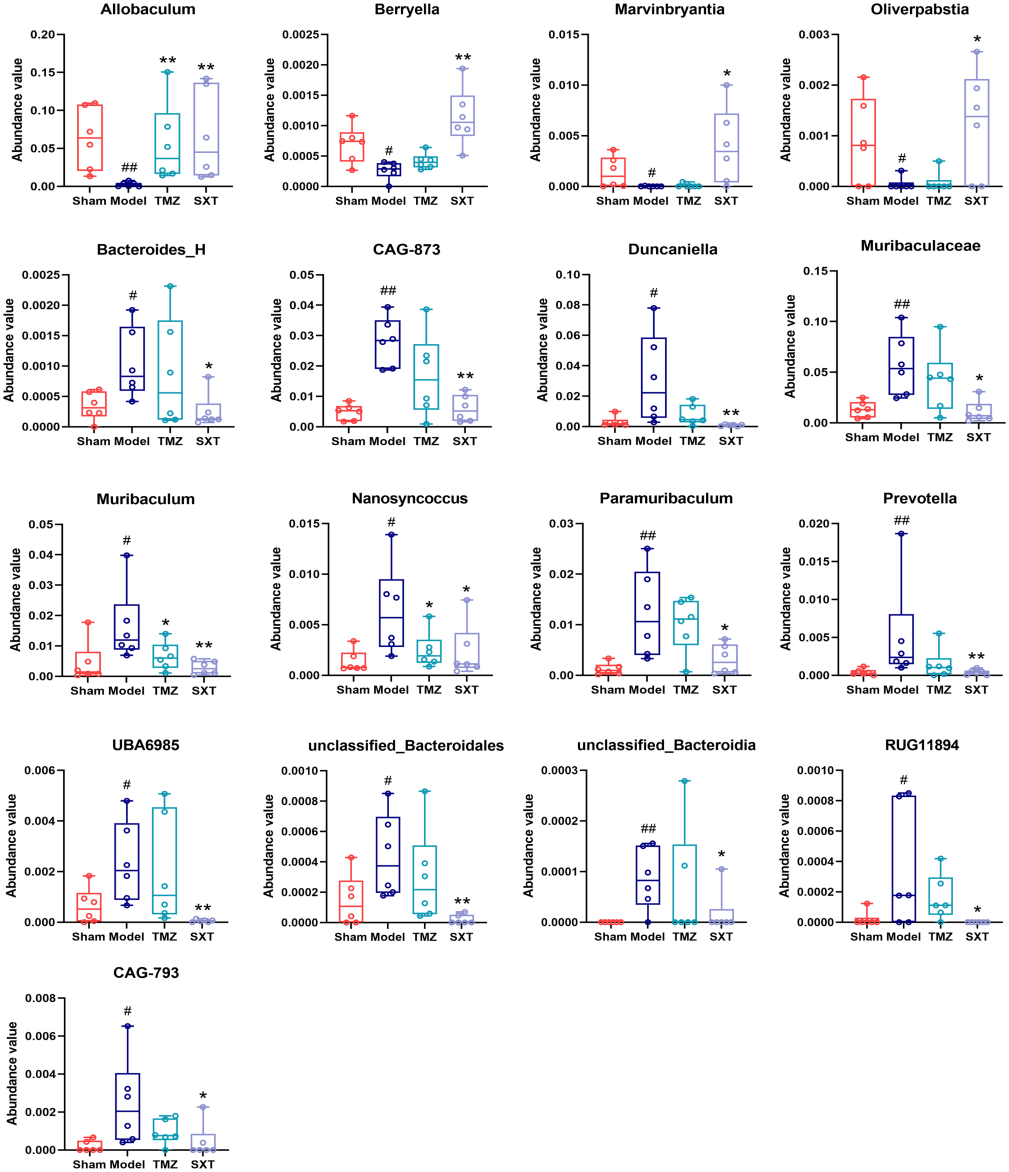
Differential analysis of gut microbiota at the genus level across four groups (*n* = 6). Differential microbiota were identified using the Kruskal–Wallis test. ^#^*p* < 0.05, ^##^*p* < 0.01 vs. the sham group; ^*^p < 0.05, ^**^p < 0.01 vs. the model group.

At the genus level, 30 genera with relative abundances exceeding 1% were identified. In the sham group, the most abundant genera (in descending order) were *Ligilactobacillus* 34.14%, *Allobaculum* 6.33%, *Lactobacillus* 5.37%, *Bifidobacterium* 4.76%, *Dubosiella* 3.62%, and *Faecalibaculum* 3.46%. In the model group, the most abundant genera were *Ligilactobacillus* 14.77%, *Lactobacillus* 12.10%, *UBA3282* 3.07%, *Duncaniella* 3.07%, *Romboutsia_B* 2.94%, and *CAG-273* 2.82%. For the TMZ group, the most abundant genera were *Ligilactobacillus* 20.70%, *Lactobacillus* 7.75%, *Desulfovibrio_R* 5.72%, *Allobaculum* 5.56%, *UBA3282* 5.14%, and *CAG-269* 3.16%. In the SXT group, the most abundant genera were *Ligilactobacillus* 20.44%, *Allobaculum* 6.57%, *Faecousia* 6.44%, *Lactobacillus* 5.53%, *Blautia_A* 5.45%, and *Romboutsia_B* 5.14% (Figure 5H, Supplementary Table S2). To further investigate the role of gut microbiota in SXT-mediated CHF treatment, genera with significantly different relative abundances between the sham and model groups were identified. Seventeen genera showing significant changes were identified following SXT treatment. Compared with the sham group, the abundances of g_*Allobaculum*, g_*Berryella*, g_*Marvinbryantia*, and g_*Oliverpabstia* were reduced in the model group but were restored following SXT treatment. Compared with the sham group, the abundances of g*_Allobaculum, g_Berryella, g_Marvinbryantia, and g_Oliverpabstia* decreased in the model group and increased after SXT intervention. However, the remaining genera displayed opposing changes (Figure 6).

#### 3.2.5 LEfSe analysis

**Figure 7:**
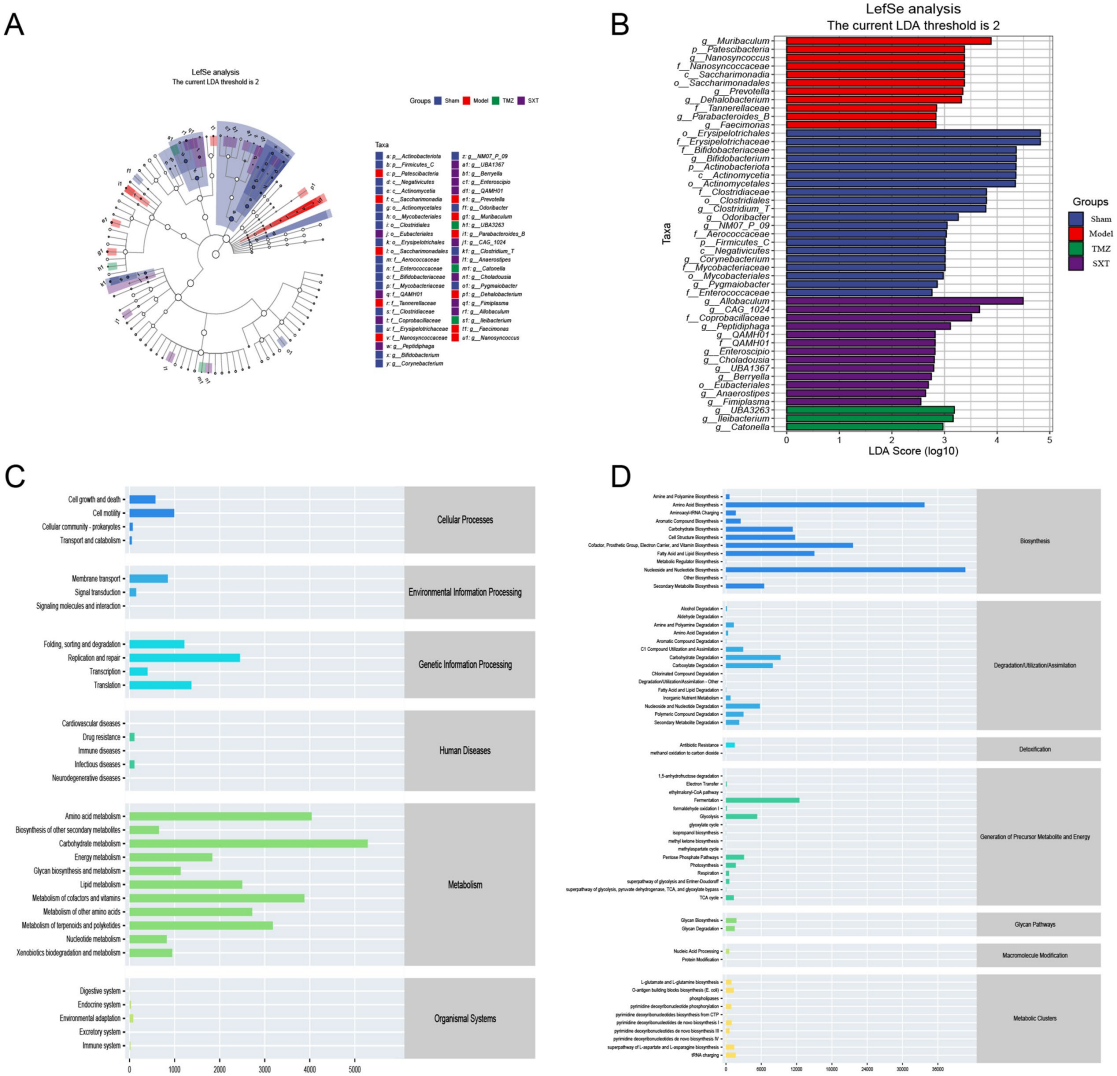
Differentially abundant taxa identified by LEfSe (*n* = 6). **(A)** Cladogram. Each circle indicates the level of classification, ranging from phylum to genus. The size of the circle reflects the abundance of gut microbiota. **(B)** Histogram. The variations in the abundance of microbiota among the four groups. Functional annotation of gut microbiota. **(C)** KEGG pathway enrichment analysis. **(D)** MetaCyc pathway enrichment analysis.

LEfSe analysis is a method that combines the Kruskal–Wallis and Wilcoxon tests with linear discriminant analysis (LDA) effect size. The analysis revealed 47 bacterial taxa as key discriminators (Figure 7A). Among these, *Erysipelotrichales* was significantly enriched in the sham group (LDA score > 4.5). Additionally, *Bifidobacteriaceae* and *Actinobacteriota* were also prominently enriched in the sham group (LDA score > 4). In the model group, *Muribaculum* was significantly enriched (LDA score > 3.5). The SXT group exhibited significant enrichment of *Allobaculum* (LDA score > 4). Previous studies have reported reduced diversity and abundance of gut microbiota in patients with cardiovascular disease and that the abundance of important gut microbiota is down-regulated (Troseid et al., 2020). Further LDA analysis showed that the sham group was enriched with 20 bacterial taxa, the model group with 11 taxa, the SXT group with 13 taxa, and the TMZ group with 3 taxa (Figure 7B). The LEfSe results are consistent with previous findings.

#### 3.2.6 Functional prediction of gut microbiota

PICRUSt2 was used to predict functional profiles of gut microbiota across groups. KEGG enrichment analysis revealed that microbial functions were predominantly associated with metabolic pathways, including carbohydrate metabolism, amino acid metabolism, cofactor and vitamin metabolism, and lipid metabolism. Genetic information processing pathways were mainly enriched in replication and repair, as well as translation. Cellular process pathways were primarily associated with cell motility, growth, and death (Figure 7C). MetaCyc enrichment analysis further supported these results, showing that microbial functions were predominantly enriched in biosynthetic processes, including nucleotide and nucleoside biosynthesis, amino acid biosynthesis, cofactor and vitamin biosynthesis, as well as fatty acid and lipid biosynthesis (Figure 7D).

### 3.3 Fecal metabolomics analysis

#### 3.3.1 Metabolic profiling

**Figure 8:**
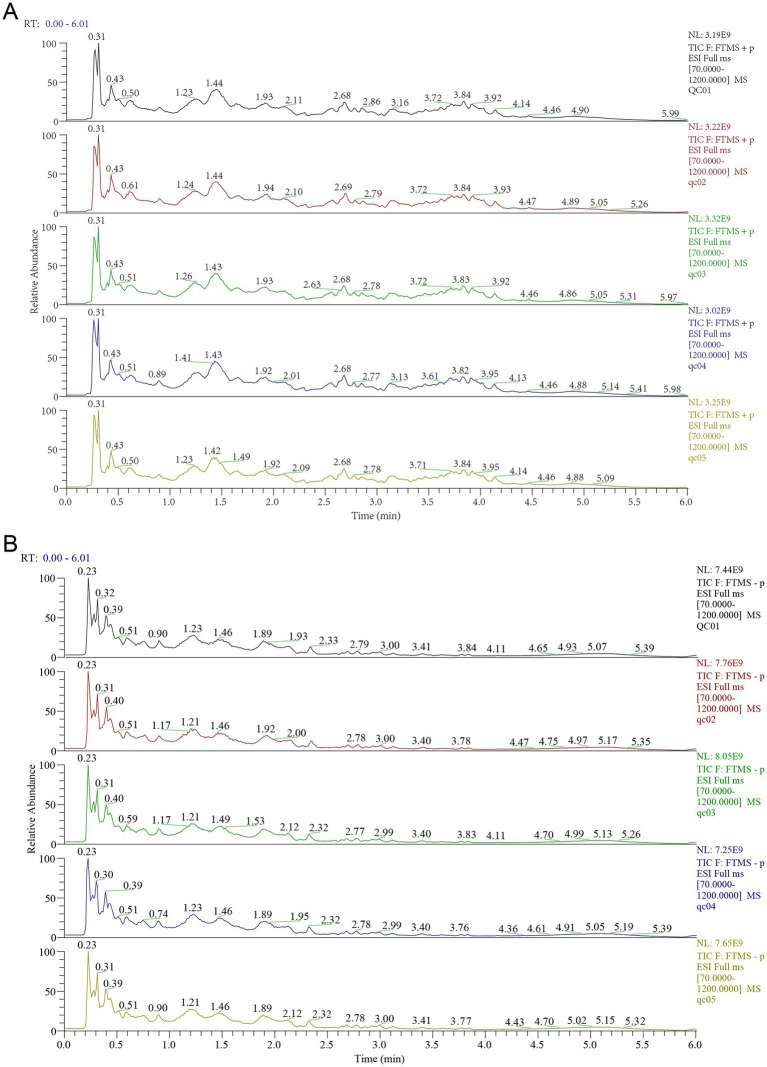
Representative chromatogram of QC samples. **(A)** Positive ion mode. **(B)** Negative ion mode.

**Figure 9:**
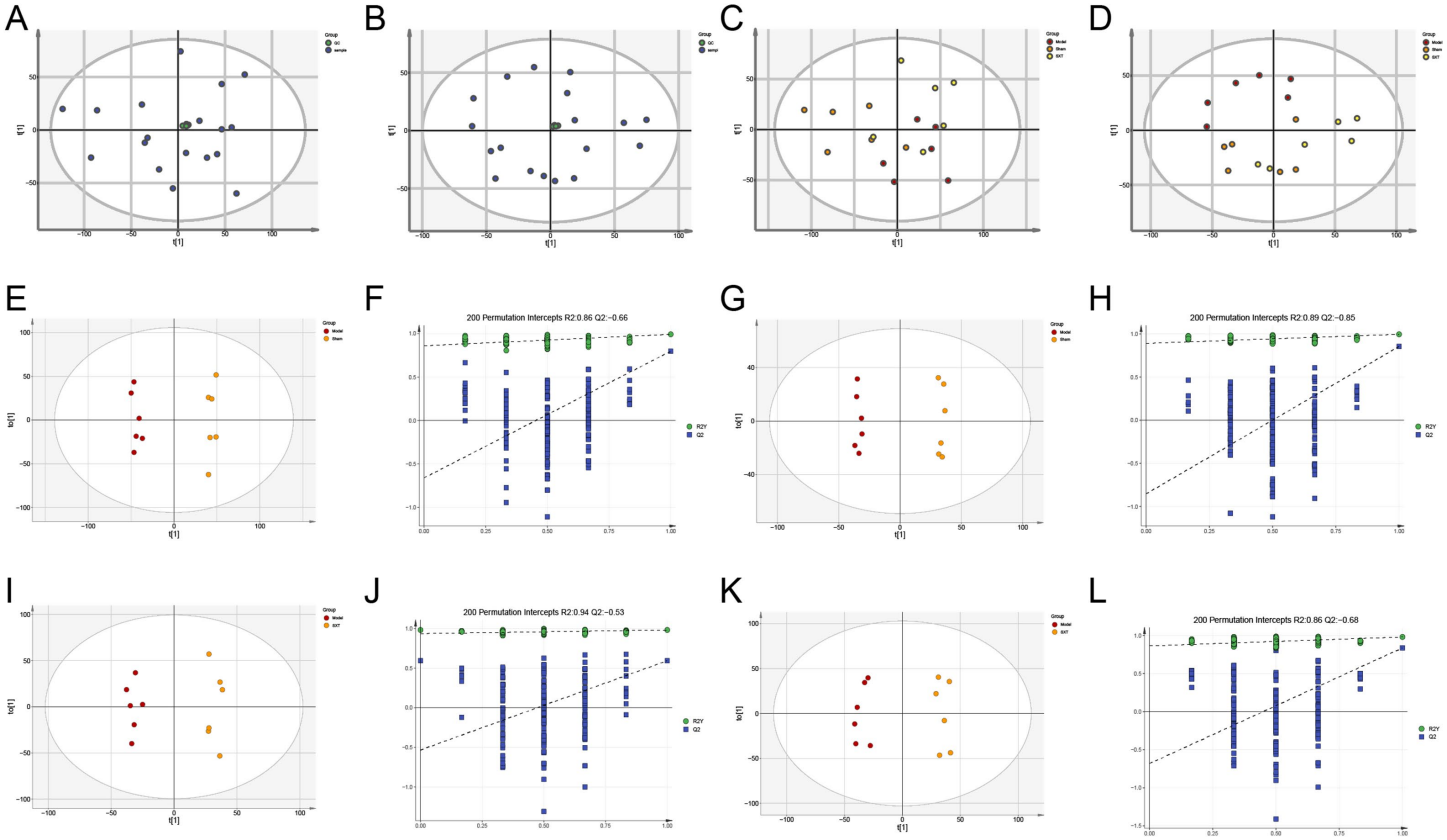
Metabolomics analysis. **(A,B)** Plots of PCA scores (with QC). **(C,D)** Plots of PCA scores for each group. OPLS-DA score plots and 200-permutation tests. **(E,F)** Positive ion pattern of the Sham vs. Model group. (R^2^X = 0.453, R^2^Y = 0.992, Q^2^ = 0.797). **(G,H)** Negative ion pattern of the Sham vs. Model group. (R^2^X = 0.302, R^2^Y = 0.996, Q^2^ = 0.856). **(I,J)** Positive ion pattern of the Model vs. SXT group. (R^2^X = 0.289, R^2^Y = 0.982, Q^2^ = 0.594). **(K,L)** Negative ion pattern of the Model vs. SXT group (R^2^X = 0.53, R^2^Y = 0.998, Q^2^ = 0.867).

The total ion chromatograms of the fecal samples are shown in Figure 8. To explore the impact of SXT on fecal metabolic differences in CHF rats, multivariate statistical analyses were performed. PCA results showed that quality control (QC) samples were tightly clustered in both positive and negative ion modes (Figures 9A,B), indicating instrument stability and data reliability. A separation trend was observed among the Sham, Model, and SXT groups in both ion modes (Figures 9C,D). In the positive ion mode, R^2^X was 0.513, while in the negative ion mode, R^2^X was 0.586. Notably, significant metabolic differences were identified between the Sham and Model groups, suggesting that TAC-induced CHF altered the fecal metabolome in rats. The metabolic profile of the SXT group exhibited a trend toward that of the Sham group, indicating that SXT may modulate intestinal metabolic alterations in CHF rats.

#### 3.3.2 Differential metabolite analysis

**Figure 10:**
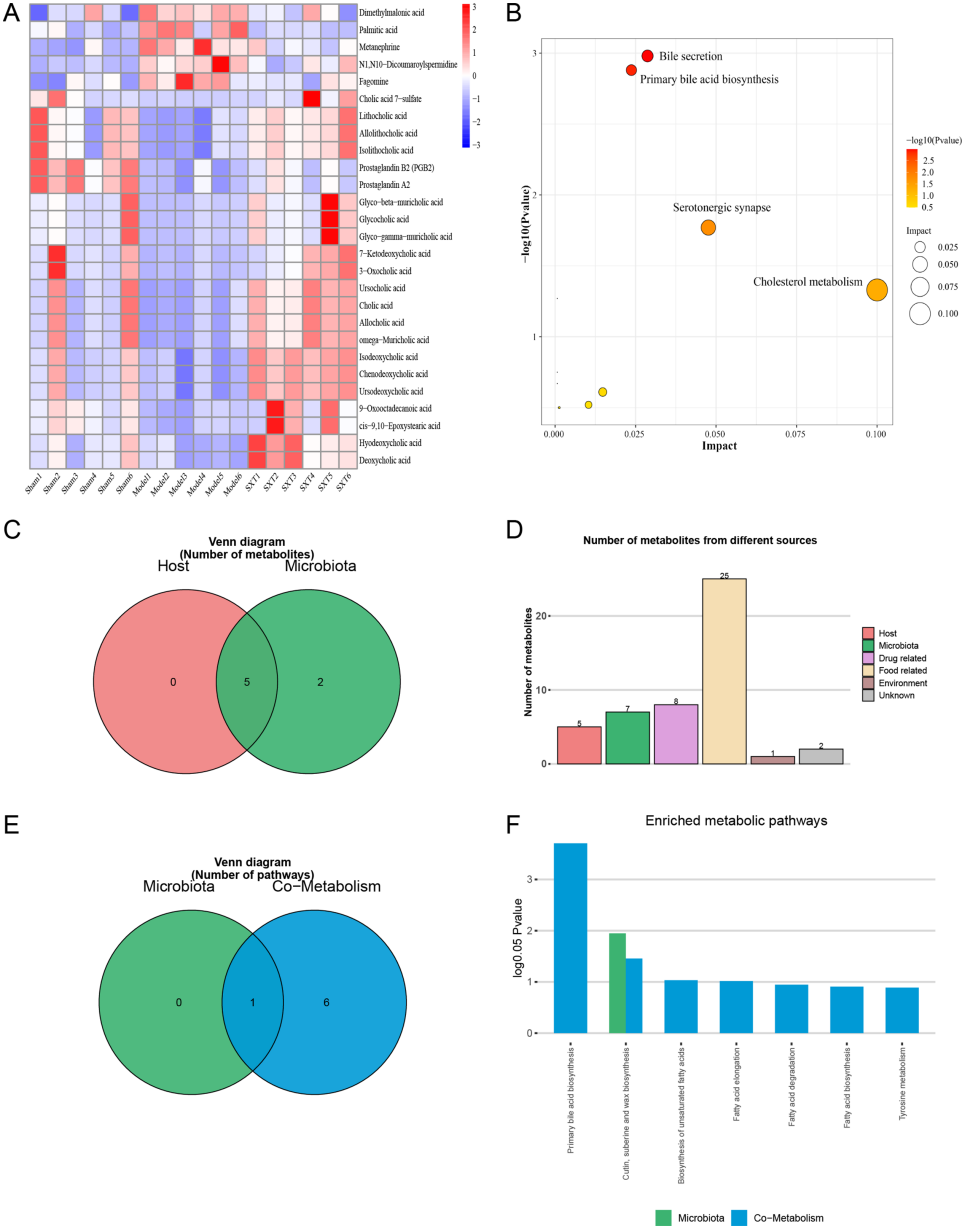
**(A)** Differential metabolites correlation heatmap. **(B)** The bubble chart of metabolic pathways. **(C)** Venn diagram of differential metabolites. **(D)** Histogram of differential metabolites. **(E)** Venn diagram of enrichment analysis of differential metabolites. **(F)** Histogram of enrichment analysis of differential metabolites.

To further investigate metabolic differences among the Sham, Model, and SXT groups, orthogonal partial least squares discriminant analysis (OPLS-DA) was performed. As shown in Figures 9E–L, clear separations were observed between the Sham and Model groups, as well as between the SXT and Model groups. The OPLS-DA model was validated through 200 permutation tests. The results showed that the Q^2^ regression line intersected the vertical axis below zero, and all *Q*^2^ values were lower than the initial *Q*^2^ point on the right. These findings indicate that the model was robust and reliable. Model parameters are provided in the legend of Figure 9. Potential biomarkers were selected based on VIP values from the OPLS-DA model (VIP > 1) and the *p*-values from the *t*-test (*p* < 0.05). A total of 27 differential metabolites were identified across the Sham, Model, and SXT groups. Metabolites are listed in Table 3. The heatmap visually illustrates the expression trends of these differential metabolites (Figure 10A). Compared with the Sham group, levels of glycocholic acid, chenodeoxycholic acid, cholic acid, and prostaglandin A2 were reduced in the Model group, whereas the levels of palmitic acid and metanephrine were increased. These metabolites showed opposite trends following SXT treatment.

**Table 3:** Differential metabolites associated with chronic heart failure in feces.

No	*m/z*	RT (s)	Formula	HMDB ID	Metabolites	lon form	VIP	Trend	
								Model/Sham	SXT/Model
1	407.2814	139.9	C_24_H_40_O_5_	HMDB0000505	Allocholic acid	NEG	1.42	↓^#^	↑^**^
2	464.3011	159.7	C_26_H_43_NO_6_	HMDB0000138	Glycocholic acid	NEG	1.68	↓^##^	↑^**^
3	438.2403	153.5	C_25_H_31_N_3_O_4_	HMDB0033469	N1, N10-Dicoumaroylspermidine	POS	1.84	↑^##^	↓^*^
4	407.2814	139.9	C_24_H_40_O_5_	HMDB0000364	Omega-muricholic acid	NEG	1.42	↓^#^	↑^**^
5	464.3011	159.7	C_26_H_43_NO_6_	HMDB0240607	Glyco-gamma-muricholic acid	NEG	1.68	↓^##^	↑^**^
6	375.2909	31.1	C_24_H_40_O_3_	HMDB0000381	Allolithocholic acid	NEG	1.51	↓^#^	↑^**^
7	405.2657	104.2	C_24_H_38_O_5_	HMDB0000391	7-Ketodeoxycholic acid	NEG	1.57	↓^#^	↑^**^
8	375.2909	31.1	C_24_H_40_O_3_	HMDB0000717	Isolithocholic acid	NEG	1.51	↓^#^	↑^**^
9	464.3011	159.7	C_26_H_43_NO_6_	HMDB0240607	Glyco-beta-muricholic acid	NEG	1.68	↓^##^	↑^**^
10	375.2909	31.1	C_24_H_40_O_3_	HMDB0000761	Lithocholic acid	NEG	1.51	↓^#^	↑^**^
11	487.2377	144	C_24_H_40_O_8_S	HMDB0002421	Cholic acid 7-sulfate	NEG	1.78	↓^##^	↑^*^
12	333.208	53	C_20_H_30_O_4_	HMDB0004236	Prostaglandin B2	NEG	1.90	↓^##^	↑^*^
13	391.2853	72.1	C_24_H_40_O_4_	HMDB0000518	Chenodeoxycholic acid	NEG	1.34	↓^#^	↑^**^
14	130.0868	195.4	C_6_H_13_NO_3_	HMDB0033453	Fagomine	POS	1.52	↑^##^	↓^*^
15	131.0351	119.3	C_5_H_8_O_4_	HMDB0002001	Dimethylmalonic acid	NEG	1.60	↑^#^	↓^*^
16	255.2335	45.2	C_16_H_32_O_2_	HMDB0000220	Palmitic acid	NEG	1.68	↑^##^	↓^**^
17	391.2853	72.1	C_24_H_40_O_4_	HMDB0000946	Ursodeoxycholic acid	NEG	1.34	↓^#^	↑^**^
18	333.208	53	C_20_H_30_O_4_	HMDB0002752	Prostaglandin A2	NEG	1.90	↓^##^	↑^*^
19	297.244	18.8	C_18_H_34_O_3_	HMDB0030979	9-Oxooctadecanoic acid	NEG	1.76	↓^##^	↑^**^
20	297.244	18.8	C_18_H_34_O_3_	HMDB0247617	cis-9,10-Epoxystearic acid	NEG	1.76	↓^##^	↑^**^
21	407.2814	139.9	C_24_H_40_O_5_	HMDB0000619	Cholic acid	NEG	1.42	↓^#^	↑^**^
22	405.2657	104.2	C_24_H_38_O_5_	HMDB0000502	3-Oxocholic acid	NEG	1.57	↓^#^	↑^**^
23	198.1134	43.1	C_10_H_15_NO_3_	HMDB0004063	Metanephrine	POS	1.68	↑^##^	↓^*^
24	391.2854	90.9	C_24_H_40_O_4_	HMDB0000733	Hyodeoxycholic acid	NEG	1.38	↓^#^	↑^**^
25	391.2853	72.1	C_24_H_40_O_4_	HMDB0002536	Isodeoxycholic acid	NEG	1.34	↓^#^	↑^**^
26	407.2814	139.9	C_24_H_40_O_5_	HMDB0000917	Ursocholic acid	NEG	1.42	↓^#^	↑^**^
27	391.2854	90.9	C_24_H_40_O_4_	HMDB0000626	Deoxycholic acid	NEG	1.38	↓^#^	↑^**^

^#^*P* < 0.05, ^##^*P* < 0.01 vs. the sham group; *P < 0.05, **P < 0.01 vs. the model group.

#### 3.3.3 Key metabolic pathway analysis of differential metabolites

Pathway enrichment analysis of identified differential metabolites was performed using MetaboAnalyst 5.0 platform. The significantly enriched pathways (*p* < 0.05) included primary bile acid biosynthesis, bile secretion, serotonergic synapse, and cholesterol metabolism (Figure 10B, Supplementary Table S3). These findings suggest that the therapeutic effects of SXT may involve multiple metabolic pathways and targets.

#### 3.3.4 MetOrigin tracing analysis of differential metabolites

**Figure 11:**
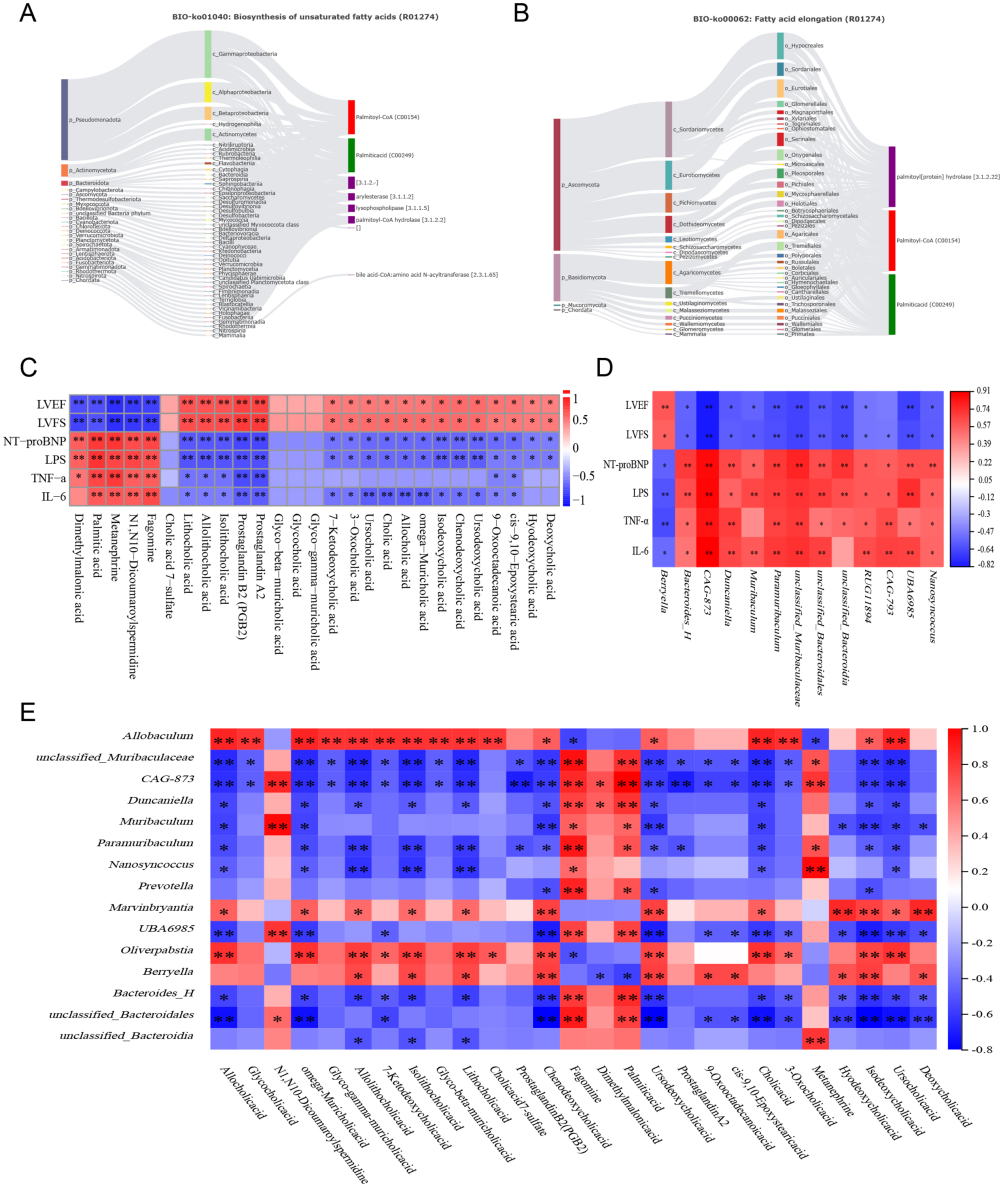
Sankey diagram of Metorigin analysis of **(A)** Biosynthesis of unsaturated fatty acids and **(B)** fatty acid elongation. **(C)** Correlation analysis between differential metabolites and clinical indicators (LVEF, LVFS, NT-proBNP, LPS, TNF-α, IL-6). **(D)** Correlation analysis between gut microbiota and clinical indicators. **(E)** Correlation analysis between differential metabolites and differential gut microbiota (*p < 0.05, **p < 0.01).

Metabolite tracing analysis identified 27 differential metabolites associated with SXT: 5 bacterial-host cometabolites, 7 bacterial metabolites, and 5 host metabolites (Figures 10C,D). Metabolite pathway enrichment analysis (MPEA) revealed that 0, 1, and 7 relevant metabolic pathways were enriched in the host, bacterial, and co-metabolic pathway databases, respectively (Figures 10E,F). Origin-based functional analysis identified primary bile acid biosynthesis, cutin, suberine, and wax biosynthesis, biosynthesis of unsaturated fatty acids, and fatty acid elongation as co-metabolic pathways between microbiota and host. A Bio-Sankey network constructed via MetOrigin analysis further visualized the biological links and statistical associations between the microbiota and metabolites, thereby facilitating a clearer illustration of the co-metabolic relationships between the microbiota and the host (Figures 11A,B).

### 3.4 Correlation analysis

#### 3.4.1 Correlation between fecal metabolites and clinical indicators

Figure 11C shows Pearson correlation analysis (|*r*| > 0.6) between fecal metabolites and clinical indicators (LVEF, LVFS, NT-proBNP, LPS, TNF-α, IL-6). The results showed that cardiac function indicators (LVEF, LVFS) were positively correlated with lithocholic acid, chenodeoxycholic acid, prostaglandin A2, prostaglandin B2, and cholic acid, while negatively correlated with palmitic acid and metanephrine, among others. In contrast, heart failure and inflammatory indicators (NT-proBNP, LPS, TNF-α, IL-6) were positively correlated with palmitic acid and metanephrine, and negatively correlated with lithocholic acid and prostaglandin A2, among others.

#### 3.4.2 Correlation between gut microbiota and clinical indicators

Figure 11D shows Pearson correlation analysis (|*r*| > 0.6) between gut microbiota and clinical indicators (LVEF, LVFS, NT-proBNP, LPS, TNF-α, IL-6). The results showed that cardiac function indicators (LVEF, LVFS) were positively correlated with *g_Berryella*, and negatively correlated with *g_Bacteroides_H*, *g_unclassified_Muribaculaceae*, and *Muribaculum*. In contrast, the correlations between heart failure and inflammatory indicators (NT-proBNP, LPS, TNF-α, IL-6) and the aforementioned bacterial genera showed opposite trends.

#### 3.4.3 Correlation between fecal metabolites and gut microbiota

Figure 11E shows Pearson correlation analysis (|*r*| > 0.6) between differential metabolites and the gut microbial genera. The results showed that bile acid metabolites (Glycocholic acid, Cholic acid, and Chenodeoxycholic acid) were significantly correlated with multiple genera. Positive correlations were observed with genera such as *Allobaculum*, *Marvinbryantia*, and *Oliverpabstia*, while negative correlations were found with *unclassified_Muribaculaceae*, *Duncaniella*, and *Bacteroides_H*. Fatty acid metabolites, such as Palmitic acid, showed positive correlations with *unclassified_Muribaculaceae*, *Duncaniella*, *Muribaculum*, and *Prevotella*, and negative correlations with *Berryella*. Prostaglandin metabolites, such as Prostaglandin A2, exhibited negative correlations with *unclassified_Muribaculaceae* and *Paramuribaculum*. Furthermore, Metanephrine was positively correlated with *unclassified_Muribaculaceae* and *Paramuribaculum*, and negatively correlated with *Allobaculum*. These findings suggest that alterations in gut microbiota composition are associated with changes in metabolic profiles.

## 4 Discussion

**Figure 12:**
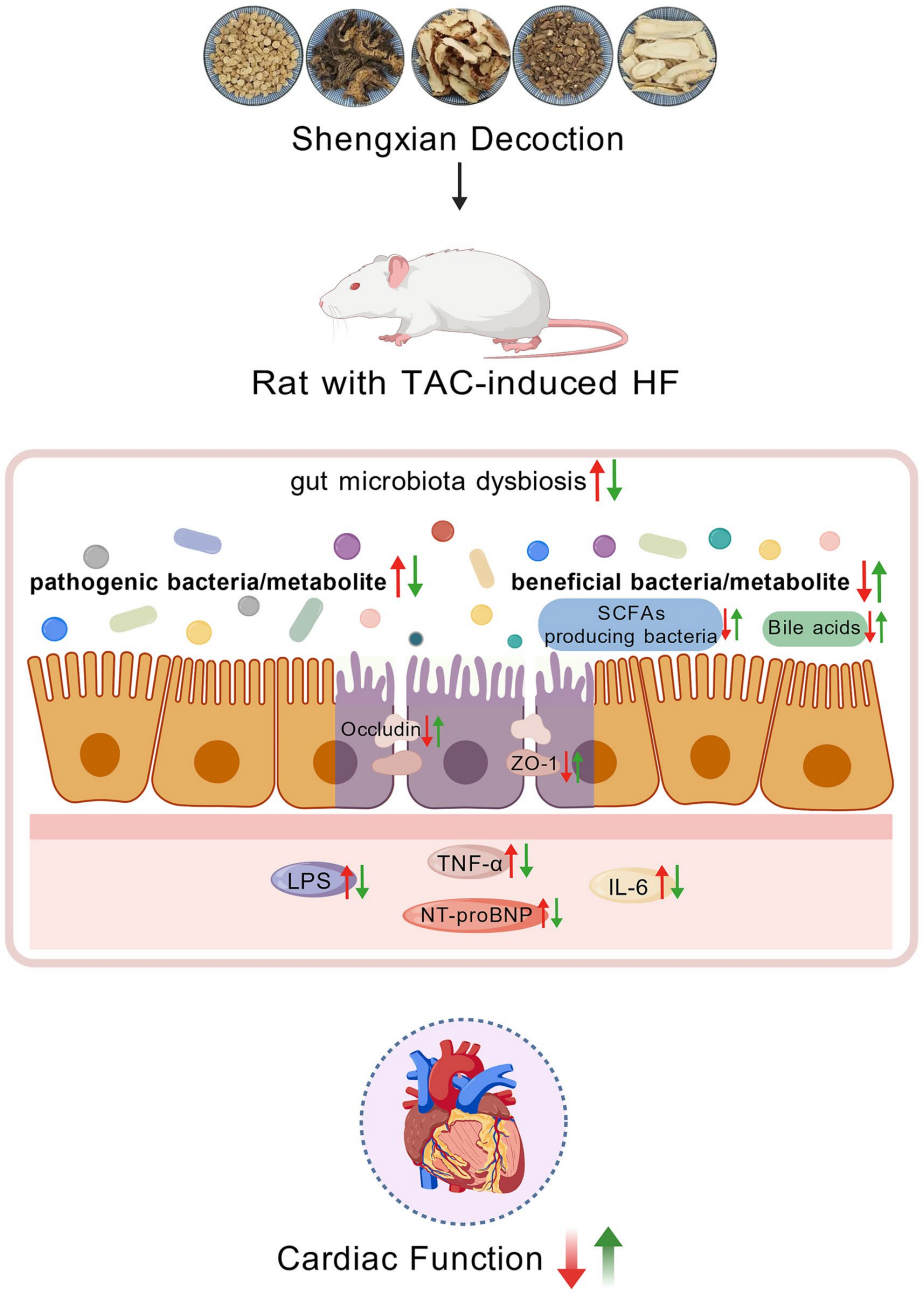
Shengxian decoction improves cardiac function in association with modulation of the gut microbiota and metabolites. This figure was created with https://www.BioGDP.com.

Heart failure is the terminal phase of numerous cardiovascular illnesses. According to the gut-heart axis hypothesis, gut microbiota and their metabolites are closely associated with heart failure (Bozkurt et al., 2021; Lupu et al., 2023). Studies have shown that the intestinal mucosa in patients with CHF exhibits ischemia, edema, and impaired barrier function, which triggers systemic inflammation and accelerates disease progression (Sandek et al., 2014). SXT is beneficial in the treatment of CHF (Yang et al., 2023). However, the regulatory mechanism of SXT in gut microbiota and its metabolites remains unclear. Figure 12 illustrates the main findings of this study.

In this study, we first assessed the extent of myocardial and intestinal injury in CHF rats and further investigated the effects of SXT intervention. The results showed that, compared with the sham group, LVEF and LVFS were decreased, while NT-proBNP levels were significantly elevated in the model group, indicating the development of heart failure. Previous studies have shown that elevated serum LPS levels are indicative of impaired intestinal barrier function. LPS can translocate into the systemic circulation through a compromised intestinal barrier and induce the production of pro-inflammatory cytokines, which may promote myocardial hypertrophy, fibrosis, and apoptosis, thereby contributing to the development and progression of CHF (Sandek et al., 2012; Jiang et al., 2019). Furthermore, previous studies have shown that increased LPS levels downregulate tight junction proteins such as occludin and ZO-1, thereby increasing intestinal permeability (Liang M. et al., 2024). Our results showed that, compared with the sham group, the model group exhibited significantly increased serum levels of LPS, TNF-α, and IL-6, accompanied by decreased expression of occludin and ZO-1. Moreover, pronounced pathological alterations were observed in both myocardial and colonic tissues in the model group. After treatment with SXT or TMZ, LVEF and LVFS were increased, serum levels of NT-proBNP, LPS, TNF-α, and IL-6 were reduced, the expression of occludin and ZO-1 was restored, and the structure of myocardial and colonic tissues was improved. However, the recovery effect of TMZ on intestinal wall damage was relatively limited. Collectively, these findings suggest that SXT exerts protective effects on both the heart and intestine in CHF rats.

Gut microbiota dysbiosis is an important feature of heart failure (Jia et al., 2020). In this study, 16S rRNA gene sequencing was used to characterize alterations in the gut microbiota. According to the Venn diagram, 345 ASVs were shared across the four groups. These ASVs may represent core microbiota involved in maintaining gut homeostasis. A total of 4,857 unique ASVs were identified in the model group, indicating that CHF development may be associated with these species. The *α* diversity analysis showed no significant differences in microbial diversity across groups. However, *β*-diversity analysis, including PCoA and NMDS, revealed significant alterations in microbial structure in CHF rats, whereas SXT or TMZ treatment partially restored microbial structure. A clinical study demonstrated no significant differences in gut microbiota *α* diversity indices, including the Shannon and Simpson indices, between healthy individuals and the HFpEF patients. However, *β*-diversity analysis revealed significant differences in microbial structure (Huang Z. Y. et al., 2021). Similarly, an animal study comparing control, heart failure, and Shenmai injection-treated rats reported no significant differences in Chao1 or Shannon indices, while *β*-diversity analysis revealed distinct microbial structures (Li et al., 2025). Collectively, our findings are consistent with previous reports, suggesting that CHF progression is associated with alterations in gut microbiota structure and that SXT may promote the restoration of gut microbial homeostasis in CHF rats.

Previous studies have shown that probiotics such as *Ligilactobacillus* can prevent heart failure and attenuate myocardial hypertrophy during myocardial infarction (Gan et al., 2014). At the genus level, *Ligilactobacillus* abundance was reduced in the model group, whereas it was increased following SXT or TMZ treatment. Additionally, the F/B ratio is a pivotal indicator of gut microbiota dysbiosis and is associated with cardiovascular disease (Magne et al., 2020). *Firmicutes* and *Bacteroidetes* contribute to enhanced substrate availability and promote intestinal carbohydrate degradation (Woting and Blaut, 2016). For instance, *Firmicutes* ferment dietary fiber to produce short-chain fatty acids (SCFAs), which provide energy for intestinal epithelial cells, reduce intestinal inflammation, and enhance host immune function (Jordan et al., 2023). A reduction in circulating SCFAs resulting from F/B ratio imbalance not only impairs intestinal energy metabolism but also contributes to cardiac hypertrophy and fibrosis (Kaye et al., 2020; Woting and Blaut, 2016). In this study, the F/B ratio at the phylum level decreased in CHF rats. However, SXT treatment significantly restored the F/B ratio. Consistent with our findings, previous studies have shown that the F/B ratio is significantly reduced in rats with adriamycin-induced heart failure and can be restored following Sini Decoction treatment (Zhao et al., 2022).

Analysis of differential bacterial genera revealed that the model group exhibited reduced abundances of *g_Allobaculum*, *g_Marvinbryantia*, *g_Berryella*, and *g_Oliverpabstia* compared with the sham group. *g_Allobaculum* is a beneficial gut bacterial genus that primarily produces SCFAs. SCFAs serve as an energy source for intestinal epithelial cells and contribute to maintaining intestinal barrier integrity (Chen et al., 2023). SCFAs reduce the secretion of pro-inflammatory cytokines, such as IL-6 and IL-8, thereby exerting anti-inflammatory effects (Li et al., 2018). *g_Marvinbryantia* belongs to the phylum *Firmicutes* and is capable of producing butyrate, a key component of microbial SCFAs. Butyrate promotes intestinal epithelial proliferation and differentiation, facilitates mucosal repair, preserves barrier integrity, and reduces bacterial and metabolite translocation, thereby mitigating inflammation (Kelly et al., 2015). Fecal butyrate concentrations in heart failure patients are negatively correlated with NT-proBNP levels (Modrego et al., 2023). Energy metabolism is critical in the progression of heart failure. Butyrate enhances mitochondrial ATP synthesis, thereby improving the contractile function of the failing heart (Panagia et al., 2020). Reduced butyrate levels are commonly observed in cardiovascular disease and are correlate with disease severity (Chakaroun et al., 2023). SCFA-producing gut bacteria may represent potential targets for preventing cardiac hypertrophy and fibrosis (Kaye et al., 2020). Restoration of gut microbiota balance and enhanced SCFA production are associated with improved prognosis in heart failure patients (Modrego et al., 2023). Correlation analysis in this study showed that the beneficial genus *g_Berryella* was positively correlated with cardiac function indices (LVEF, LVFS) and negatively correlated with NT-proBNP, LPS, TNF-α, and IL-6. SXT treatment increased the abundance of the aforementioned bacterial genera. These bacterial genera may represent important taxa negatively associated with CHF progression and may contribute to delaying disease progression in rats.

In addition, the abundances of potentially pathogenic genera, such as *g_Bacteroides_H*, *g_unclassified_Muribaculaceae*, *Muribaculum*, and *g_Prevotella*, were increased in the model group. *g_Bacteroides_H* belongs to the phylum *Bacteroidetes* and has been positively associated with an increased risk of cardiovascular disease (Lisewski et al., 2020). A previous study reported that benzene exposure reduced intestinal tight-junction proteins occludin and Claudin-1 in mice, accompanied by increased abundance of *unclassified_Muribaculaceae* (Wang et al., 2023). *Muribaculum*, considered a pathogenic bacterium, is associated with inflammatory responses (Zhou et al., 2023). *Prevotella* is a mucin-degrading bacterial genus that may induce intestinal mucosal inflammation (Larsen, 2017). Compared with healthy individuals, hypertensive patients exhibit reduced gut microbial abundance and diversity, with a significant increase in *Prevotella* (Li et al., 2017). Correlation analysis showed that pathogenic genera such as *g_Bacteroides_H*, *g_unclassified_Muribaculaceae*, and *Muribaculum* were positively correlated with NT-proBNP, LPS, TNF-α, and IL-6, and negatively correlated with cardiac function indices (LVEF and LVFS). After SXT treatment, the abundances of the aforementioned pathogenic genera were markedly reduced, whereas TMZ regulated only a subset of these bacteria. Our analysis revealed that TAC-induced CHF induces gut microbiota dysbiosis, consistent with previous reports (Boccella et al., 2021). SXT may attenuate CHF progression by alleviating intestinal inflammation and restoring gut homeostasis.

The gut microbiota can influence host functions by modulating host or microbial metabolites (Li et al., 2024). Accumulating evidence indicates that gut microbial metabolites play a critical role in CHF pathogenesis (Kitai and Tang, 2018). Compared with metabolic profiles of biological fluids such as serum and urine, fecal metabolomics better captures the integrated effects of host genetics, environment, and diet, providing more comprehensive metabolic information (Brown et al., 2022). Therefore, fecal samples from the sham, model, and SXT groups were analyzed metabolomically to evaluate the regulatory effects of SXT on gut microbiota and associated metabolites in CHF rats. A total of 27 differential metabolites were identified across the three groups. Metabolomic analysis showed that in TAC-induced CHF rats, levels of glycocholic acid, chenodeoxycholic acid, cholic acid, lithocholic acid, and prostaglandin A2 were decreased, whereas palmitic acid and metanephrine levels were elevated. SXT treatment dramatically reversed these metabolic perturbations, restoring the metabolic profile toward normal levels. Pathway enrichment analysis of differential metabolites identified several pathways, including primary bile acid biosynthesis, bile secretion, serotonergic synapse, and cholesterol metabolism. Although the impact values of these pathways were modest, the *p* values were statistically significant, indicating biological relevance. These results suggest that the therapeutic effects of SXT may involve multi-target metabolic regulation.

Bile acids form a crucial chemical barrier in the intestine and are synthesized in the liver via cholesterol catabolism. Gut microbiota, such as *Bacteroides* and *Clostridium*, convert primary bile acids into secondary bile acids, while changes in bile acid composition modulate gut microbiota distribution (Sayin et al., 2013). Bile acids suppress the proliferation of pathogenic bacteria and protect the intestine from microbial invasion (Grüner and Mattner, 2021). Furthermore, bile acids facilitate the absorption of dietary fats, lipid-soluble compounds, and cholesterol (Wan et al., 2020). Certain bile acids function as steroid-like signaling molecules and exert cardioprotective effects by modulating cardiac inflammation, heart rate, and vascular tone (Mayerhofer et al., 2017; Zhou et al., 2025). Dysregulated bile acid metabolism can induce myocardial injury, including hypertrophy and cardiomyocyte apoptosis (Vasavan et al., 2018). Collectively, bile acids are essential for maintaining intestinal homeostasis and regulating cardiovascular function. Clinical studies indicate that patients with CHF exhibit reduced primary bile acid levels, accompanied by an elevated secondary-to-primary bile acid ratio. This alteration correlates with overall patient survival (Mayerhofer et al., 2017). In the study, metabolites enriched in the primary bile acid biosynthesis pathway included glycocholic acid, chenodeoxycholic acid, and cholic acid. Compared with the sham group, these bile acids were significantly reduced in the model group but were restored following SXT treatment.

Prostaglandin A2 (PGA2) is an endogenous metabolite derived from arachidonic acid (AA). AA is a long-chain polyunsaturated fatty acid, and its metabolic processes play a critical role in the development and progression of CHF (Ma et al., 2023). Moreover, AA-derived metabolites regulate diverse physiological and pathological processes such as apoptosis, cellular metabolism, and vascular function (Zhou et al., 2021). Palmitic acid acts as a ligand for Toll-like receptor 4 (TLR4), activating TLR4-mediated inflammatory signaling and inducing cardiomyocyte injury by promoting the release of pro-inflammatory cytokines, including IL-6 and TNF-α (Su et al., 2025; Zheng et al., 2020). Metanephrine is an intermediate metabolite of catecholamine metabolism. Elevated catecholamine levels are associated with increased cardiovascular risk (Zhou et al., 2020). In this study, compared to the sham group, prostaglandin A2 levels were decreased, whereas palmitic acid and metanephrine levels were increased in the model group; SXT treatment reversed these alterations. These results suggest that SXT may attenuate CHF progression by modulating the gut metabolite profile.

Correlation analysis showed that cardiac function indices, including LVEF and LVFS, were positively correlated with chenodeoxycholic acid, cholic acid, and prostaglandin A2, and negatively correlated with palmitic acid and metanephrine. In contrast, heart failure-related and inflammatory indices, including NT-proBNP, LPS, TNF-α, and IL-6, exhibited inverse correlation patterns with these metabolites. These findings suggest that SXT may improve cardiac function and attenuate inflammatory responses by upregulating beneficial metabolites while downregulating deleterious metabolites. Moreover, these key metabolites may serve as potential biomarkers for assessing therapeutic efficacy.

The gut microbiota and host engage in close crosstalk via substrate co-metabolism and metabolite exchange. Source-based functional analysis identified several key host–microbe co-metabolic pathways, including primary bile acid biosynthesis, cutin, suberin and wax biosynthesis, unsaturated fatty acid biosynthesis, and fatty acid elongation. Furthermore, 16S rRNA gene-based functional prediction revealed that gut microbial functions were predominantly enriched in carbohydrate metabolism, amino acid metabolism, cofactor and vitamin metabolism, and lipid metabolism. Correlation analysis between differential metabolites and gut microbiota indicated that bile acid and fatty acid metabolites are associated with specific bacterial genera. For example, glycocholic acid was positively associated with *g_Allobaculum*, whereas lithocholic acid was positively associated with *g_Allobaculum* and *g_Oliverpabstia*, but negatively associated with *g_unclassified_Muribaculaceae* and other genera. Palmitic acid was positively associated with *g_unclassified_Muribaculaceae*, *g_Duncaniella*, and other genera. Overall, significant differences were observed in both gut microbiota composition and metabolic profiles between heart failure and normal rats. This “microbiota-metabolism” dysregulation may contribute to CHF pathogenesis. SXT modulates the abundance of specific gut microbial taxa and associated metabolites in CHF rats, restoring both to a normal state. These findings provide a foundation for future research on the pathological processes related to gut microbiota metabolism in CHF.

SXT exhibits potential to improve cardiac function and modulate the gut microbiota and associated metabolites. However, several limitations remain. First, only male SD rats were used, and a non-sham control group was not included, which may limit the comprehensive assessment of sex-differential effects and the distinction between surgical influences and baseline physiological states. Furthermore, it remains unclear whether the amelioration of dysbiosis and metabolite abnormalities is a consequence of improved cardiac function or whether they play a critical role in the progression of heart failure. This requires further investigation and validation. To address these limitations, future studies will include animals of different sexes and incorporate a non-sham control group. Moreover, fecal microbiota transplantation combined with advanced analytical approaches will be employed to further investigate the relationship between gut microbiota alterations and heart failure.

## 5 Conclusion

In conclusion, SXT ameliorates gut microbiota dysbiosis and associated metabolic disorders in CHF rats, providing a theoretical foundation for elucidating the association of gut microbiota and their metabolites with CHF.

## Data Availability

The raw data generated in this study have been submitted to the NCBI SRA (https://www.ncbi.nlm.nih.gov/) under accession number PRJNA1226280.

## Footnotes

1
https://metacyc.org/

2
https://www.kegg.jp/
