# Supplementary material for: Shengxian decoction modulates gut microbiota and microbial metabolism in rats with chronic heart failure
Source: Front Microbiol. 2026 May 20;17:1791537. doi: 10.3389/fmicb.2026.1791537 (PMC13232063; doi:10.3389/fmicb.2026.1791537)
Supplement: Supplementary file 1 [file Supplementary_file_1.zip › Supplementary Materials.DOCX]

Supplementary Material

## LC-MS analysis of SXT

## Chromatographic conditions

Separation was performed using a Waters UPLC I-Class ultra-performance liquid chromatography system with the following conditions: column: Waters UPLC HSS T3 (1.8 μm, 2.1 mm × 100 mm); mobile phases: A: 0.1 % formic acid aqueous solution, B: acetonitrile; the gradient elution program was detailed in Table S1. The flow rate was 0.3 mL/min, the injection volume was 10 µL, and the column temperature was maintained at 40°C.

## Mass spectrometric conditions

Mass spectrometry analysis was performed using a quadrupole-orbitrap ion trap mass spectrometer (Q Exactive™) equipped with an electrospray ionization (ESI) source. The ion source parameters were set as follows: the ion source voltages were 3.7 kV in positive ion mode and 3.5 kV in negative ion mode; the capillary heater temperature 320℃; the sheath gas pressure was 30 psi, and auxiliary gas pressure was 10 psi; the vaporizer temperature was 300°C; The sheath gas, auxiliary gas, and collision gas were all nitrogen, with the collision gas pressure set as 1.5 mTorr. The full scan parameters were as follows: resolution of 70,000, automatic gain control target 1 × 10⁶, maximum isolation time 50 ms, and m/z scan range 100–1500. Compound identification was conducted using a data-dependent MS² (dd-MS²) scan mode. The system control and data acquisition were performed using Xcalibur 2.2 SP1.48 software.

Table S1 Gradient elution table

| Time (min) | Mobile phase | |
| --- | --- | --- |
|  | A (v%) | B (v%) |
| 0 | 98 | 2 |
| 1.0 | 98 | 2 |
| 41.0 | 0 | 100 |
| 50.0 | 0 | 100 |
| 50.1 | 98 | 2 |
| 52.0 | 98 | 2 |

**
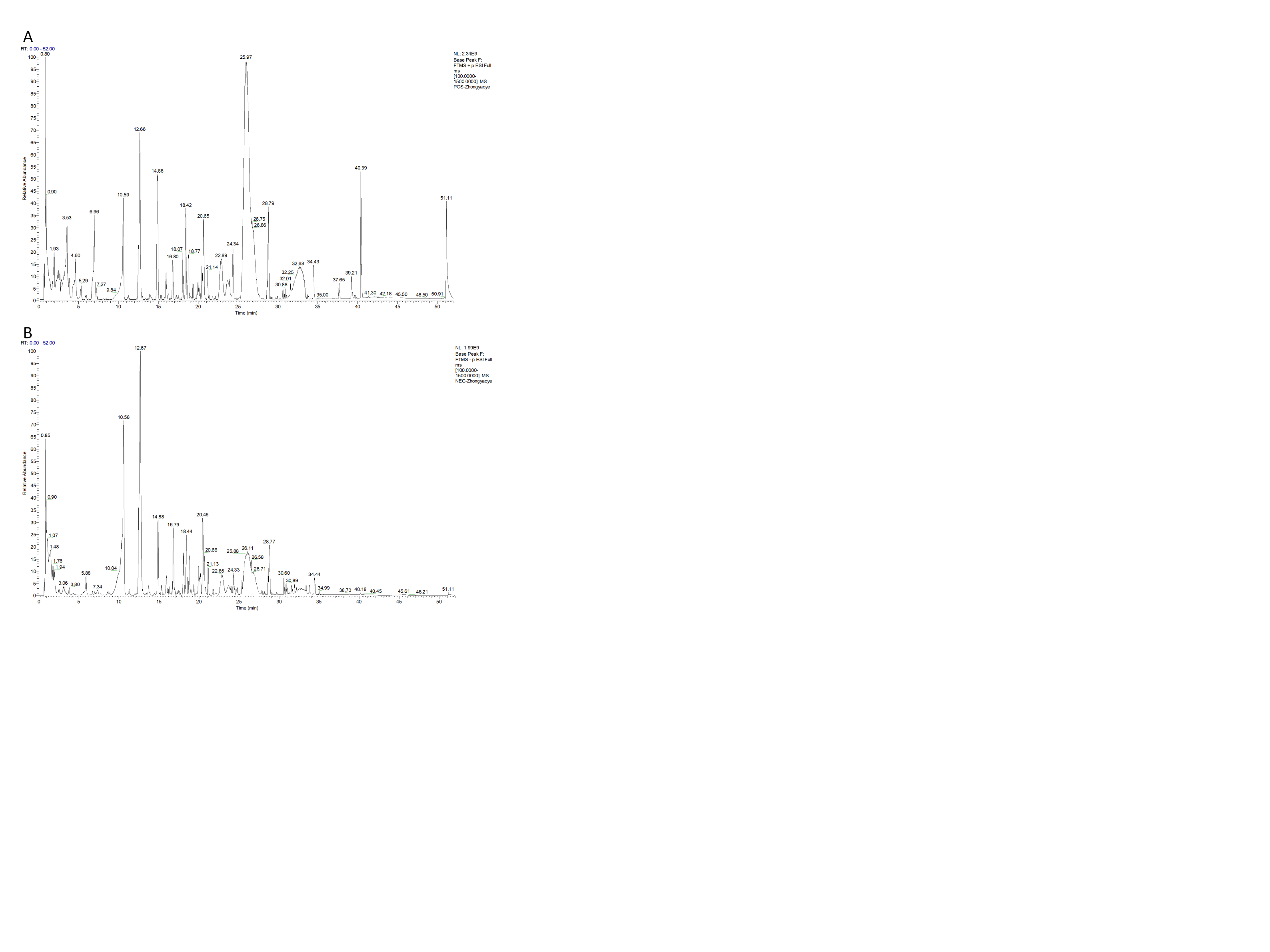
**

**Supplementary Figure 1.** Chemical profiling of SXT. (A) Base peak chromatograms of SXT in ESI (+) and ESI (-) modes.

Table S2 Key chemical constituents identified in SXT by LC-MS analysis

| **No.** | **RT**  **(min)** | **Compound name** | **Molecular**  **formula** | **M/Z** | **Adduct** | **Source** |
| --- | --- | --- | --- | --- | --- | --- |
| 1 | 0.90 | Proline | C₅H₉NO₂ | 116.071 | [M+H]⁺ | Astragali Radix |
| 2 | 0.92 | 1-O-α-D-glucopyranosyl-β-D-fructofuranose | C₁₂H₂₂O₁₁ | 360.150 | [M+H]⁺ | Cimicifugae Rhizoma |
| 3 | 5.88 | Piscidic acid | C₁₁H₁₂O₇ | 255.051 | [M-H]⁻ | Cimicifugae Rhizoma |
| 4 | 9.62 | Chlorogenic acid | C₁₆H₁₈O₉ | 355.102 | [M+H]⁺ | Astragali Radix |
| 5 | 10.59 | Neomangiferin | C₂₅H₂₈O₁₆ | 585.144 | [M+H]⁺ | Anemarrhenae Rhizoma |
| 6 | 11.28 | Foliamangiferoside A | C_20_H_22_O_10_ | 421.115 | [M-H]⁻ | Anemarrhenae Rhizoma |
| 7 | 12.66 | Mangiferin | C₁₉H₁₈O₁₁ | 423.091 | [M+H]⁺ | Anemarrhenae Rhizoma |
| 8 | 13.91 | Ferulic acid | C₁₀H₁₀O₄ | 177.054 | [M+H]⁺ | Cimicifugae Rhizoma |
| 9 | 14.84 | Isoferulic acid | C₁₀H₁₀O₄ | 177.054 | [M+H]⁺ | Cimicifugae Rhizoma |
| 10 | 14.86 | Calycosin-7-O-  β-D-glucoside | C₂₂H₂₂O₁₀ | 447.127 | [M+H]⁺ | Astragali Radix |
| 11 | 15.48 | Astraisoflavan glucoside | C_23_H₂₄O₁₁ | 477.139 | [M+H]⁺ | Astragali Radix |
| 12 | 15.94 | Isomangiferin | C₁₉H₁₈O₁₁ | 423.091 | [M+H]⁺ | Anemarrhenae Rhizoma |
| 13 | 16.01 | Saikoisoflavonoside A | C_28_H₃₂O₁₄ | 637.178 | [M-H]⁻ | Bupleuri Radix |
| 14 | 16.62 | Lobetyolin | C_26_H_38_O₁₃ | 581.219 | [M+H]⁺ | Platycodonis Radix |
| 15 | 16.80 | 2,4',6-Trihydroxy-4-  methoxybenzophenone | C₁₄H_12_O₅ | 261.075 | [M+H]⁺ | Anemarrhenae Rhizoma |
| 16 | 17.61 | Isoquercitrin-7,2'-di-  O-glucoside | C_29_H_38_O_15_ | 644.254 | [M+H]⁺ | Astragali Radix |
| 17 | 18.92 | Isorhamnetin-3-O-  β-D-rutinoside | C₂₈H₃₂O₁₆ | 623.162 | [M-H]⁻ | Bupleuri Radix |
